# Salicylic acid modulates secondary metabolism and enhanced colchicine accumulation in long yellow daylily (*Hemerocallis citrina*)

**DOI:** 10.1093/aobpla/plae029

**Published:** 2024-05-21

**Authors:** Yeminzi Miao, Hanmei Li, Junjie Pan, Binxiong Zhou, Tianjun He, Yanxun Wu, Dayun Zhou, Weimin He, Limin Chen

**Affiliations:** Lishui Institute of Agricultural and Forestry Sciences, 827 Liyang Stress, Lishui, Zhejiang 323000, China; College of Forestry Science and Technology, Lishui Vocational & Technical College, Lishui, Zhejiang 323000, China; Lishui Institute of Agricultural and Forestry Sciences, 827 Liyang Stress, Lishui, Zhejiang 323000, China; Lishui Institute of Agricultural and Forestry Sciences, 827 Liyang Stress, Lishui, Zhejiang 323000, China; Lishui Institute of Agricultural and Forestry Sciences, 827 Liyang Stress, Lishui, Zhejiang 323000, China; Lishui Science & Technology Bureau, Lishui, Zhejiang 323000, China; Lishui Institute of Agricultural and Forestry Sciences, 827 Liyang Stress, Lishui, Zhejiang 323000, China; Lishui Institute of Agricultural and Forestry Sciences, 827 Liyang Stress, Lishui, Zhejiang 323000, China; Lishui Institute of Agricultural and Forestry Sciences, 827 Liyang Stress, Lishui, Zhejiang 323000, China

**Keywords:** candidate gene, colchicine, *Hemerocallis citrina*, phytoregulator, salicylic acid, transcriptomics

## Abstract

Abstract. Salicylic acid (SA) is an essential phytoregulator that is widely used to promote the synthesis of high-value nutraceuticals in plants. However, its application in daylily, an ornamental plant highly valued in traditional Chinese medicine, has not been reported. Herein, we investigated the exogenous SA-induced physiological, transcriptional and biochemical changes in long yellow daylily (LYD). We found that 2 mg/L foliar SA treatment significantly improved LYD plant growth and yield. Transcriptome sequencing and differentially expressed genes (DEGs) analysis revealed that the phenylpropanoid biosynthesis, isoquinoline alkaloid biosynthesis, sulfur metabolism, plant hormone signal transduction and tyrosine metabolism were significantly induced in SA-treated leaves. Many transcription factors and antioxidant system-related DEGs were induced under the SA treatment. Biochemical analyses showed that the leaf contents of soluble sugar, soluble protein (Cpr), ascorbic acid (AsA) and colchicine were significantly increased by 15.15% (from 30.16 ± 1.301 to 34.73 ± 0.861 mg/g), 19.54% (from 60.3 ± 2.227 to 72.08 ± 1.617 mg/g), 30.45% (from 190.1 ± 4.56 to 247.98 ± 11.652 μg/g) and 73.05% (from 3.08 ± 0.157 to 5.33 ± 0.462 μg/g), respectively, under the SA treatment. Furthermore, we identified 15 potential candidate genes for enhancing the growth, production and phytochemical content of LYD. Our results provide support for the bioaccumulation of colchicine in yellow daylily and valuable resources for biotechnological-assisted production of this important nutraceutical in *Hemerocallis spp*.

## Teaser

This study reveals the exogenous salicylic acid (SA)-induced physiological, transcriptional and biochemical changes in long yellow daylily (LYD). It found that 2 mg/L foliar SA treatment could be applied to improve LYD plant growth, yield, and accumulation of soluble sugar, soluble protein, ascorbic acid and colchicine. LYD is a potential material for biotechnological-assisted production of edible colchicine.

## Introduction

Long yellow daylily (LYD, *Hemerocallis citrina*) is a monocotyledonous perennial herbaceous plant from the family Liliaceae (Qing *et al.* 2021). It is an important vegetable and ornamental plant with tremendous applications as a functional food, flavouring agent and raw material in traditional Chinese medicine (Guo *et al.* 2022, 2023). LYD is widely distributed in Mongolia, Russia, China, Japan, Korea and Europe and is popular for its attractive flowers (Guo *et al.* 2022, 2023). It is a material of choice in treating depression and has also shown antioxidant, anti-inflammatory, anti-constipation, anti-lactation deficiency and neuroprotective abilities (Tian *et al.* 2017; Matraszek-Gawron *et al.* 2019; Zhong *et al.* 2021; Ma *et al.* 2022, 2023; Jiang *et al.* 2023; Liang *et al.* 2023). It is a rich source of nutrients (amino acids, carbohydrates, phosphorus, calcium, iron, and vitamins C, B1, B2 and B5) and functional substances, such as lecithin, alkaloids, phenolic acids, flavonoids and saponins (Guo *et al.* 2022; Li *et al.* 2022). With its important ornamental and medicinal potentials, enhancing the production and nutraceutical content of LYD will contribute to its sustainable use and value addition.

Colchicine (an alkaloid) is one of the most ancient remedies used in diverse medical disciplines, such as dermatology, immunology, oncology, cardiology and nephrology. (Solak *et al.* 2017; Dasgeb *et al.* 2018; Robinson and Chan 2018; Siak *et al.* 2021; Huber *et al.* 2023). Since ancient times, colchicine has been the most effective drug for the treatment of neutrophilic inflammation, mainly gout, amyloidosis and familial Mediterranean fever (Richette and Bardin 2010; Dalbeth, Lauterio and Wolfe 2014; Leung, Yao Hui and Kraus 2015; Dasgeb *et al.* 2018; Schattner 2022). It is also effective in treating cancers (Kumar, Sharma and Mondhe 2016), acute pericarditis (Imazio *et al.* 2013; Siak *et al.* 2021; Huet *et al.* 2022), atherosclerosis (Meyer-Lindemann *et al.* 2022) and coronary diseases (COVID-19) (Nidorf *et al.* 2020; Mikolajewska *et al.* 2021). Besides its high medicinal value, colchicine is an important compound used in biotechnology to induce plant diploidization and polyploidization (Ślusarkiewicz-Jarzina *et al.* 2017; Tammu, Nuringtyas and Daryono 2021; Wu *et al.* 2022; Bajpai and Chaturvedi 2023). As for many plant-derived nutraceuticals, colchicine production still relies on natural resources from which it is extracted. Studies revealed that it is biosynthesized in several herbaceous species members of the Liliaceae family from phenylalanine and tyrosine (Nett, Lau and Sattely 2020; Nett and Sattely 2021; Stander, Papon and Courdavault 2021). Unfortunately, it is produced in very low quantities in whole plants. Therefore, enhancing its availability in source materials is of great interest. The report by Traub *et al.* indicated that *Hemerocallis* species may contain an appreciable amount of colchicine (Traub 1949). We thus speculated that *H. citrina* could represent a source of edible colchicine production for medical and biotechnological applications.

SA is a vital plant regulator that affects diverse growth and development processes, including seed germination, pigment accumulation, stomatal movements, photosynthesis, heat production, ethylene biosynthesis, enzyme activities, nutrient uptake, abscission reversal, membrane functions, flower induction and metabolic activities (Larqué-Saavedra 2007; Ali 2021). Owing to its hormone activity, SA has been widely applied to enhance plants’ abiotic and biotic stress tolerance and to promote secondary metabolite biosynthesis and accumulation (Larqué-Saavedra 2007; Ali 2021; Li, Sun and Liu 2022; Liu *et al.* 2022; Monteiro *et al.* 2022). It has been applied to improve the content of anthocyanins and polyphenols in grapevine (Oraei *et al.* 2019; Blanch, Gómez-Jiménez and del Castillo 2020); induce flavonoid synthesis in wheat (Gondor *et al.* 2016); stimulate alkaloid accumulation in *Arthrospira platensis* (Hadizadeh *et al.* 2019); promote glucosinolates accumulation in *Brassica oleracea* (Yi *et al.* 2016) and improve the quality of blueberry fruits (Jiang *et al.* 2022b, 2022a). Hence, we hypothesized that exogenous application of SA may improve LYD growth and production, and enhance the synthesis and accumulation of nutrients, colchicine and other secondary metabolites. Understanding the physiological, biochemical and molecular changes associated with SA applications in LYD, will offer fundamental resources for improving the crop’s performance and quality.

The main objective of the present study was to reveal the physiological, biochemical and transcriptional changes associated with exogenous SA treatment of LYD. We determined the optimal concentration of SA to improve LYD growth, development and yield. We investigated the impact of the optimal SA concentration on nutrients (soluble sugar and soluble protein), antioxidant enzymes, flavone, total phenolic, ascorbic acid and colchicine contents of LYD leaves. Furthermore, we performed a comparative transcriptomics analysis and unveiled significantly induced pathways and potential candidate genes. Our findings provide fundamental resources for biotechnological-assisted edible colchicine production in LYD.

## Materials and Methods

### Plant material and experimental procedures

LYD seedlings preserved by the Lishui Institute of Agricultural and Forestry Sciences were used in this study. The roots were transplanted into a nutrient bowl (height 17 cm, diameter 20 cm) and cultivated in an incubator (temperature 28 ± 2 °C, 14 h light/10 h dark and humidity 50-60%). When the seedlings reached 8–10 cm in height, they were transplanted into pots (one individual plant per pot) in an insect-free greenhouse and allowed to grow for 20 days until the 5-leaf stage. Uniform seedlings with healthy plants were selected and divided for further experimentation. The day/night temperatures and relative humidity in the greenhouse were 24 ± 1 °C/20 ± 1 °C and 60 ± 5%, respectively. The diameter and height of the pot were 20 cm and 30 cm, respectively. Six treatments, including CK (control), T1 (0.5 mg/L), T2 (1 mg/L), T3 (2 mg/L), T4 (4 mg/L) and T5 (6 mg/L), with six replications were set. All plants were watered normally. SA was dissolved in dimethyl sulfoxide and diluted as needed to the desired concentration. Tween-20 (1 mL) was added to each 1 L of aqueous solution and sprayed on the leaves. After 14 days following the foliar SA treatment, physiological indicators such as plant height, leaf length, and leaf width were measured and samples (the middle part of the three leaves) were collected for biochemical indicators and transcriptomics analyses. The total chlorophyll content was assessed on three fully opened leaves using a SPAD metre with three technical measures per leaf. After the yellow cauliflower flower buds grew, commercial flower buds were collected for yield-related traits measurement. Samples for biochemical traits and transcriptomics analysis were directly frozen in liquid nitrogen and kept at −80 °C until used.

### Evaluation of biochemical indicators

Based on the results of growth and yield indicators, only the CK and T3 groups were selected for biochemical and transcriptome sequencing. Eight biochemical traits, including antioxidant enzymes (SOD and CAT), soluble sugar (SS), soluble protein (Cpr), total phenols, total flavone, reduced ascorbic acid (AsA) and colchicine were selected to explore SA-induced metabolic changes. All biochemical tests were performed with three biological and technical replications.

For the evaluation of Cpr, SS and the activity of CAT (catalase) and SOD (superoxide dismutase), 100 mg of each sample were ground in a pre-cooled mortar with liquid nitrogen and extracted with 1.5 mL phosphate buffer (1 mM EDTA, 10 mM cysteine and pH 7.5). Then, all extracts were collected separately and centrifuged (10 000 g for 15 min). The Cpr was evaluated using the bicinchoninic acid assay (BCA) method. In brief, 4 μL of the extract and 200 μL of BCA working solution were mixed. After incubation at 60 °C for 30 min, the absorbance was recorded at 562 nm using a microplate reader (SpectraMax ABS plus, Molecular Devices, CA, USA). The extraction buffer was used as a control, and the bovine serum albumin was used as standard (*y* = 4.2274*x*−0.3374, *R*^2^ = 0.9962). The results were expressed per mg of fresh weight (mg/g FW). The SS assay kit was used for soluble sugar content assessment (Buysse and Merckx 1993). Similarly, the activities of CAT and SOD were measured using their respective specific kits (Yan *et al.* 2023).

For the evaluation of total phenols content, total flavonoid content and reduced ascorbic acid (AsA) content, 100 mg frozen samples were extracted with 10 mL of ethanol and water (80: 20 v/v) at 37 °C for 2 hr. Next, the extracts were centrifuged at 5000 g for 20 min. The total flavonoids was determined using the NaNO_2_–AlCl_3_–NaOH method (Luan *et al.* 2023). The total phenols was evaluated using the Folin Phenol biochemical kit (Sun *et al.* 2023a). The AsA content was evaluated using the red phenanthroline colorimetric method (plant ascorbic acid content detection kit) (Sun *et al.* 2023b). All the test kits were obtained from Norminkoda Biotechnology Co., Ltd. Wuhan, China.

### Evaluation of colchicine content

Colchicine extraction and quantification were performed following the method described by Al-Fayyad *et al.* (2002). The samples were dried and ground to powder. Then 15 g was extracted with methanol–water (1: 8) at 30°C for 24 h, repeated five times. The crude extracts were pooled, filtered, and centrifugated (12 000 g for 15 min). Next, the supernatants were evaporated to dryness at 55 °C. The residues were dissolved in 5% acetic acid, followed by extraction with petroleum ether to remove non-alkaloid compounds. The aqueous acid residues were further extracted with ethyl ether, followed by a pH adjustment to 9 with ammonium hydroxide. Finally, the aqueous extracts were extracted with chloroform, followed by the evaporation of the chloroform to dryness. The residues were weighted, and the extracts were reconstituted with methanol–water for HPLC (high-performance liquid chromatography) analysis using an Agilent 1260. The HPLC conditions were as follows: Column, PL1512-5501 ChromSphe C18, 250 mm × 4.6 mm, 5 μm; flow rate, 0.5 mL/min; Injection volume, 10 μL; mobile phase, methanol–water 50:50; detector wavelength, 350 nm. Pure colchicine (C9754) was purchased from Sigma–Aldrich (St. Louis, MO, USA) for calibration.

### RNA extraction, library construction, sequencing and alignment

Total RNA from leaf samples was extracted with a Trizol reagent kit (Invitrogen, Carlsbad, CA, USA) as per the manufacturer’s specifications. The genomic DNA was discarded using DNase I (TaKara, Beijing, China). RNA quality was investigated on an Agilent 2100 Bioanalyzer (Agilent Technologies, Palo Alto, CA, USA) and quantified using the ND-2000 (NanoDrop Technologies). Only high-quality RNA (OD260/280 = 1.8–2.2, OD260/230 ≥ 2.0, RIN ≥ 6.5, 28S:18S ≥ 1.0, > 1μg) samples were used for sequencing library construction using TruSeqTM RNA sample preparation Kit (Illumina, San Diego, CA). After qualified mRNA fragmentation, cDNAs were constructed using NEB (Next Ultra RNA Library Prep Kit, Ipswich, MA, USA), and adapters were ligated. The resulting cDNA library was sequenced on the Illumina sequencing platform (HiSeq xten/NovaSeq6000 sequencer). SeqPrep (https://github.com/jstjohn/SeqPrep) and Sickle (https://github.com/najoshi/sickle) software were used to check the quality of raw paired-end. The clean reads were aligned to the *H. citrina* reference genome (Qing *et al.* 2021) by the HISAT2 (http://ccb.jhu.edu/software/hisat2/index.shtml) software (Kim, Langmead and Salzberg 2015). Finally, we assembled the mapped reads using StringTie (http://www.string-db.org/) (Pertea *et al.* 2015).

### Differentially expressed genes (DEGs) and functional enrichment analysis

The expression level of transcripts was computed according to the transcripts per million reads (TPM) method, and RSEM (http://deweylab.biostat.wisc.edu/rsem/) was used to quantify each gene abundance (Li and Dewey 2011). DEGs analysis was carried out using the DESeq2 software (Love, Huber and Anders 2014) at FDR (false discovery rate) ˂0.05 and |fold change| ≥ 1. KEGG (Kyoto Encyclopedia of Genes and Genomes, http://www.genome.jp/kegg/kaas) and GO (Genes Ontology, http://geneontology.org/) enrichment analyses were achieved using KOBAS (2.0) and GO seq software, respectively. Significant enrichment terms were screened out at *P*-value <0.05.

### Quantitative RT–PCR analysis

The RNA was extracted from leaf samples using a modified CTAB method (Kanani *et al.* 2019). Reverse transcription (RT) was conducted with the Monad 1st Strand cDNA Synthesis Kit, and the qRT-PCR analysis was achieved using Tb Green® Premix Ex Taq™ II (Takara, Beijing, China) as previously described (Huang *et al.* 2022, 2023). All samples had three biological and technical replicates. Actin gene (*J01298*) was used as an internal control (Hou *et al.* 2017) to normalize the expression levels of target genes via the 2^-ΔΔCT^ method (Livak and Schmittgen 2001). The NCBI’s primer designing tool, PRIMER-BLAST software was used to design specific primers for each gene (see Supporting Information—Table S3).

### Data analysis

Excel 2016 software, GraphPad Prism v9 (GraphPad Software Inc., La Jolla, CA, USA), SR plots (Tang *et al.* 2023) and R (version 4.3) were used for data processing and graph construction. Principal component analysis (PCA) analysis was carried out using the R package prcomp. Analysis of variance (ANOVA) and post hoc test (Tukey test) were performed for multiple comparisons at *P* < 0.05. For statistical differences between CK and T3, a *t*-test was performed and the significance was set at *P* < 0.05. Heatmaps were constructed using TBtools-II software (Chen *et al.* 2023).

## Results

### Impact of exogenous salicylic acid on *H. citrina* growth and yield

**Figure 1.**
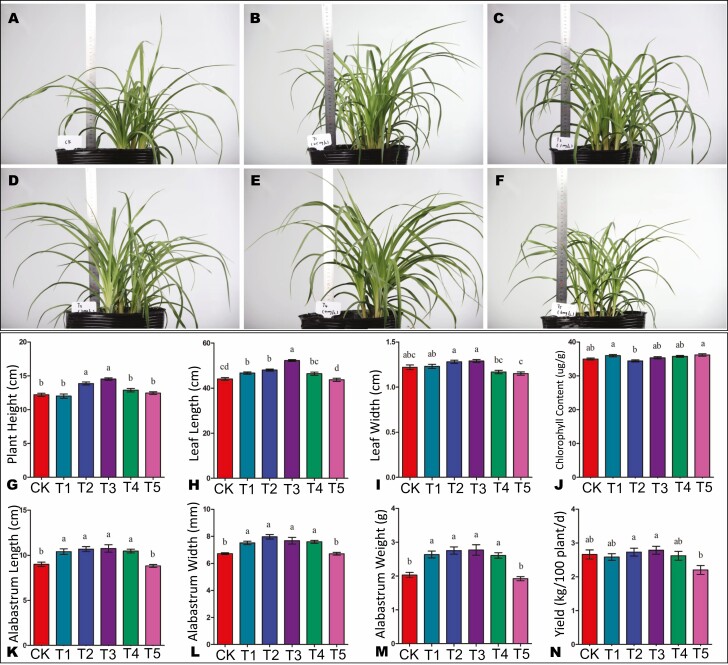
(A)–(F) Morphology of control plant (A, CK) and SA-treated plants. B–F represent different SA treatments of T1 (0.5 mg/L), T2 (1 mg/L), T3 (2 mg/L), T4 (4 mg/L) and T5 (6 mg/L), respectively. (G)–(N) Variation in plant height, leaf length, leaf width, chlorophyll content, alabastrum length, alabastrum wide, alabastrum weight and yield, respectively, of CK and different SA treatments. Different later above bars indicate statistical difference at *P* ˂ 0.05.

To explore the impacts of exogenous SA application on the growth and production of *H. citrina* plants, we proceeded to morphological observations and investigated the variation in agronomic traits under different SA treatments, including T1 (0.5 mg/L), T2 (1 mg/L), T3 (2 mg/L), T4 (4 mg/L) and T5 (6 mg/L). The morphologies of the plants 14 days after SA treatment are shown in Fig. 1A–F. In general, low SA concentrations (0.5–2 mg/L) improved the growth and yield parameters, whereas high SA concentration (>4 mg/L) showed opposite effects (Fig. 1). The growth and yield traits were significantly improved under T3 than under other treatments (Fig. 1). For instance, the plant height, leaf length, leaf width, alabastrum length, alabastrum wide, alabastrum weight and yield were significantly increased under T3 compared to the control (CK) and other treatments (Fig. 1G–I, K–N). No major differences were observed in the chlorophyll content (Fig. 1J). These results showed that T3 was the optimal SA concentration to promote growth and higher yield in *H. citrina*. Therefore, we selected T3 for further analyses.

### Salicylic acid-induced higher accumulation of colchicine, ascorbic acid, soluble protein and soluble sugar

**Figure 2.**
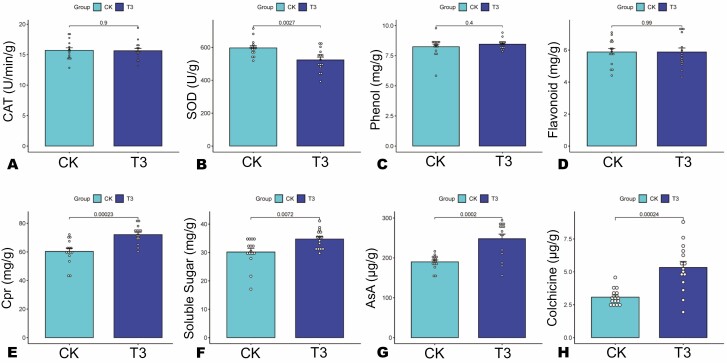
Variation in biochemical traits between the control (CK) and the optimal SA treatment, T3 (2 mg/L). (A) Catalase activity; (B) Superoxide dismutase activity; (C) Total phenols content; (D) Total flavone content; (E) Soluble protein content; (F) Soluble sugar content; (G) reduced ascorbic acid content; and (H) Colchicine content. Significant difference was set at *P* ˂ 0.05. The *P*-value of each comparison is shown at the top of the bar graphs.

To reveal the SA-induced biochemical changes in *H. citrina*, we evaluated the activity of antioxidant enzymes (CAT and SOD) and the content of soluble protein (Cpr), soluble sugar, reduced ascorbic acid (AsA), total phenol, flavone and colchicine in CK and T3 leaves (Fig. 2). No significant difference in CAT activity between CK and T3 was recorded, whereas the activity of SOD in CK was significantly higher than that in T3 (Fig. 2A and B). The total phenol and flavone contents of CK and T3 were also statistically identical (Fig. 2C and D). Regarding the other biochemical traits, the SA application induced a significant increase in the content of Cpr, soluble sugar, AsA and colchicine in T3 compared to CK (Fig. 2E–H). For instance, the soluble sugar, Cpr, AsA and colchicine content in *H. citrina* leaves were increased by 15.15, 19.54, 30.45 and 73.05 %, respectively. Of them, colchicine content was the most significantly increased, with an improvement from 3.08 ± 0.157 μg/g (CK) to 5.33 ± 0.462 μg/g (T3) (Fig. 2H). The leaf SS, Cpr and AsA contents significantly increased from 30.16 ± 1.301 to 34.73 ± 0.861 mg/g, 60.3 ± 2.227 to 72.08 ± 1.617 mg/g and 190.1 ± 4.56 to 247.98 ± 11.652 μg/g, respectively (Fig. 2E–G).

### Comparative transcriptome sequencing and DEGs

**Figure 3.**
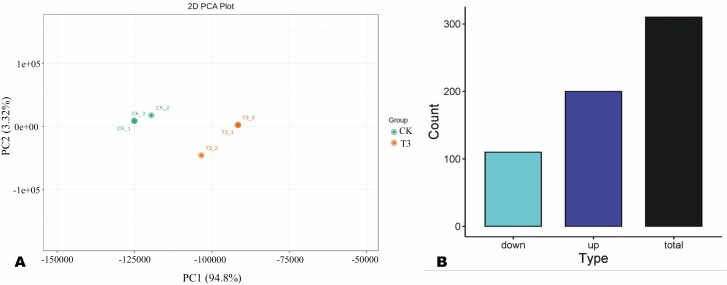
(A) Principal component analysis (PCA) of samples based on TPM values of all genes. (B) Number differentially expressed genes (DEGs) between CK and T3.

To insights into the SA-induced molecular changes in *H. citrina*, CK and T3 leaf samples were subjected to transcriptome sequencing. The RNA sequencing yielded 38 467 202 to 88 292 204 bp of raw reads, with clean reads ranging from 37 765 600 to 88 088 068 bp (see Supporting Information—Table S1). The Q20, Q30 and GC content varied from 96.46 to 97.59 %, 90.09 to 92.98 % and 45.8 to 47.91%, respectively (see Supporting Information—Table S1), indicating the high quality of the RNA-seq data. The total mapping rates against the reference genome were 79.81–85.95 %, respectively (see Supporting Information—Table S1). PCA of samples showed that the transcriptomes of CK and T3 were different and could be discriminated by PC1 of 94.8 % (Fig. 3A).

**Figure 4.**
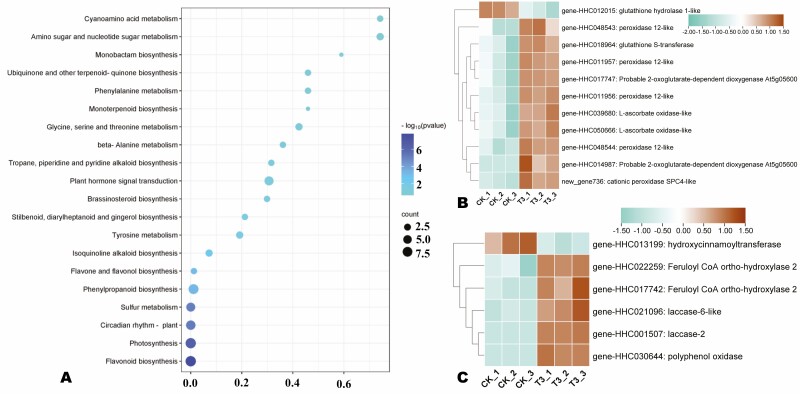
(A) KEGG annotation and enrichment results of DEGs between CK and T3. (B) Expression patterns of DEGs related to antioxidant system. (C) Expression patterns of DEGs related to phenylpropanoid biosynthesis. The key is located on the right-hand side in each case.

To examine transcriptional changes under T3 treatment, we screened out all DEGs. A total of 310 DEGs were identified, including 200 significantly induced in T3 (Fig. 3B and see Supporting Information—Table S2). The volcano plot of DEGs is presented in see Supporting Information—Fig. S1. To unveil the key molecular mechanisms affected by the SA treatment, we carried out GO and KEGG annotation and enrichment analyses of DEGs. The general GO result is presented in see Supporting Information—Fig. S2. The most induced GO terms related to biological processes were flavonoid metabolic process, protein-chromophore linkage, response to Karrikin and sulphate metabolism (see Supporting Information—Fig. S2A). Regarding cellular components, intrinsic and integral components of membrane, membrane part, extracellular regions and external encapsulating structure were the main enriched (see Supporting Information—Fig. S2B). Meanwhile, in the molecular function, the DEGs were mostly assigned to cofactor binding, cation binding, metal ion binding, and oxidoreductase activity (see Supporting Information—Fig. S2C). KEGG enrichment revealed that the DEGs were primarily involved in photosynthesis, circadian rhythm-plant, flavonoid biosynthesis, phenylpropanoid biosynthesis, sulfur metabolism, plant hormone signal transduction and isoquinoline alkaloid biosynthesis (Fig. 4A).

### Influence of salicylic acid on antioxidant system and phenylpropanoid biosynthesis

**Figure 5.**
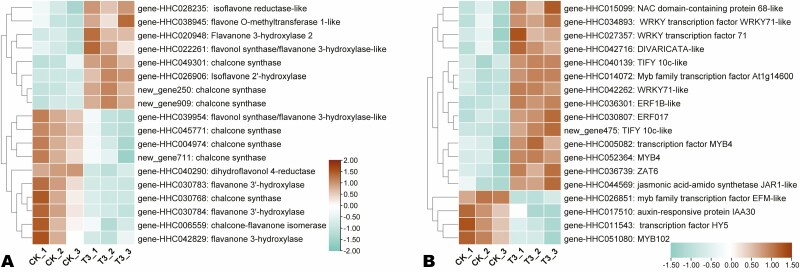
Expression patterns of DEGs related to flavonoid biosynthesis (A), and transcription factors/phytohormones (B). The key is located on the right-hand side in each case.

The KEGG analysis indicated that the SA application modulated the antioxidant system and phenylpropanoid metabolism. Therefore, we explored the expression patterns of DEGs related to the antioxidant system, flavonoid biosynthesis, phenylpropanoid biosynthesis and tyrosine metabolism (Figs. 4B, C and 5A). Most of the DEGs related to the antioxidant system, including peroxidases, glutathione S-transferase and ascorbate oxidase were up-regulated under T3 (Fig. 4B). Five out of six DEGs related to phenylpropanoid biosynthesis/isoquinoline alkaloid biosynthesis, including laccase 2 (*gene-HHC001507*), polyphenol oxidase (*gene-HHC030644*), etc., were induced under T3 (Fig. 4C). Regarding flavonoid biosynthesis, 18 DEGs were screened out, including eight up-regulated and 10 down-regulated under the SA treatment (Fig. 5A).

### Transcription factors (TFs) and phytohormone-related DEGs

TFs and phytohormones are critical for plant growth and developmental processes. We identified 16 and two TF and phytohormone-related DEGs, respectively, and examined their expression patterns under CK and T3 (Fig. 5B). Thirteen of the TF-related DEGs, including one NAC (*gene-HHC015099*), three WRKYs (*gene-HHC027357*, *gene-HHC034893*, and *gene-HHC042262*), three MYBs (*gene-HHC014072, gene-HHC005082* and *gene-HHC052364*), two ERFs (*gene-HHC030807* and *gene-HHC036301*), two TIFYs (*gene-HHC040139* and *new_gene475*), one ZAT (*gene-HHC036739*) and one DIVARICATA (*gene-HHC042716*) were induced under the SA treatment (Fig. 5B). Regarding the phytohormone-related DEGs, only *gene-HHC044569*, encoding a jasmonic acid-amido synthetase was up-regulated under T3 (Fig. 5B).

### Potential candidate genes and qRT-PCR validation

Candidate genes are essential for the molecular-assisted breeding and biotechnology perspectives. To identify potential candidate genes for targeted improvement of *H. citrina* plant growth, yield and content of nutraceuticals, we focused on pathways that were significantly induced under T3. We screened out a total of 15 potential candidate genes, including one polyphenol oxidase (*gene-HHC030644*), two transcription factors WRKY71-like (*gene-HHC042262* and *gene-HHC034893*), two ethylene-responsive factors (*gene-HHC030807* and *gene-HHC036301*), two peroxidase 12-likes (*gene-HHC011956* and *gene-HHC048544*), etc. (Table 1). Of them, *new_gene521* (a new gene with unknown function) was the most significantly induced under T3 (Table 1).

**Table 1.** List of the potential candidate genes and their annotation

Gene_ID	|Log2FC|	Nr Annotation
*new_gene521*	13.263	NA
*gene-HHC030644*	8.367	XP_010910928.1^PREDICTED: polyphenol oxidase, chloroplastic^57.25%
*gene-HHC040478*	7.818	XP_021676207.1^germin-like protein subfamily 1 member 17^64.36%
*gene-HHC001507*	6.798	XP_020098212.1^laccase-2^79.38%
*gene-HHC026906*	6.268	AGI98133.1^cytochrome P450 CYP81^44.13%
*gene-HHC022259*	4.048	XP_020092748.1^protein DMR6-LIKE OXYGENASE 2^70.37%; Feruloyl CoA ortho-hydroxylase 2
*gene-HHC042262*	3.989	XP_020252240.1^WRKY transcription factor WRKY71-like^58.08%, Short = OsWRKY76^47.78%
*gene-HHC034893*	3.882	XP_020265728.1^WRKY transcription factor WRKY71-like^51.51%, Short = OsWRKY71^45.67%
*gene-HHC030807*	2.842	XP_004287662.1^ethylene-responsive transcription factor ERF017^53.15%
*gene-HHC036301*	2.422	XP_020248108.1^ethylene-responsive transcription factor 1B-like^64.25%, Short = AtERF1B
*gene-HHC038101*	1.883	XP_020257154.1^primary amine oxidase 1-like^75.23%
*gene-HHC011956*	1.753	XP_020107531.1^peroxidase 12-like^79.32%, Short = Atperox P12
*gene-HHC048544*	1.721	XP_020107531.1^peroxidase 12-like^81.99%, Short = Atperox P12
*new_gene736*	1.605	XP_020246461.1^cationic peroxidase SPC4-like^85.37%
*gene-HHC044569*	1.39	XP_020272199.1^jasmonic acid-amido synthetase JAR1-like^73.5%; Short = OsGH3-5

**Figure 6.**
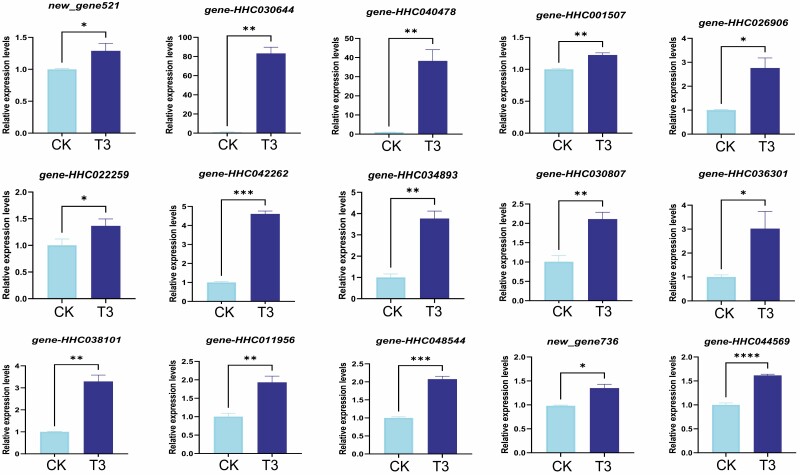
qRT-PCR validation of the RNA-seq data and potential candidate genes. *, **, ***, and **** indicate significantly different at *P* < 0.05, 0.01, 0.001 and 0.0001, respectively.

To validate the RNA-seq data and confirm the potential candidate genes, they were subjected to qRT-PCR analysis. As shown in Fig. 6, the qRT-PCR results confirmed that these genes were significantly up-regulated under T3 compared to CK.

## Discussion

Improving plant growth and content of higher-medicinal values functional compounds is essential to sustain their use in human health. Thus, the present study showed the potential of foliar SA treatment to enhance the growth, yield, ascorbic acid and colchicine content of *H. citrina*. Moreover, the involved molecular mechanisms were revealed through comparative transcriptomics analysis.

SA is a critical signal molecule that modulates plant immunity and growth processes through interplay with other phytohormones to regulate cell division, expansion and metabolism (Li, Sun and Liu 2022). It has been applied at low concentrations to enhance the growth, development and productivity of many horticultural plants, such as *Capsicum chinense* and *Brosimum alicastrum* (Tucuch-Haas *et al.* 2017). Research on wheat and maize has also demonstrated the positive effects of exogenous SA on plant growth, development and productivity under diverse environmental conditions (El-Mergawi and Abd El-Wahed 2020; Mohammed *et al.* 2023). As per previous reports in other plants, we found that low SA concentrations (0.5–2 mg/L) improved the growth and yield parameters of *H. citrina*, whereas high SA concentration (>4 mg/L) showed opposite effects. Particularly, the plant height, leaf length, leaf width, alabastrum length, alabastrum wide, alabastrum weight and yield were significantly increased under 2 mg/L (T3) SA treatment compared to the control (CK) and other treatments. Moreover, we recorded a significant increase in the content of Cpr, soluble sugar, AsA, and colchicine under T3 compared to CK. These results indicate that 2 mg/L is the optimal SA concentration to induce signalling mechanisms toward growth, production and quality improvement in *H. citrina*. Concordantly, functional analysis of DEGs revealed that sulphur metabolism and plant hormone signal transduction (TFs and phytohormone-related DEGs) were significantly induced under T3 treatment. Sulphur metabolism is critical for all organisms, as sulphur is required for the biosynthesis of antioxidants, sulpholipids, amino acids (cysteine and methionine), secondary metabolites, proteins, SAM (S-adenosylmethionine, precursor of ethylene) and cofactors (De Kok *et al.* 2012). The antioxidant system-related DEGs were up-regulated under T3. These findings suggest that 2 mg/L SA application might improve *H. citrina* plant tolerance capability to stresses. Taken together, the above results infer that 2 mg/L foliar SA application could be recommended to promote growth, production, and quality improvement in *H. citrina*.

Colchicine is a pharmacologically active tricyclic alkaloid used for various medical applications (Solak *et al.* 2017; Dasgeb *et al.* 2018; Robinson and Chan 2018; Siak *et al.* 2021; Huber *et al.* 2023). However, an uncontrolled increase in colchicine concentration can lead to toxicity and death due to overdose (Krishna and Shivankar 2021). The presence of colchicine in LYD has been associated with poisoning issues (Traub 1949). Accordingly, Tang *et al.* and Qing *et al.* have performed a series of analyses regarding LYD containing colchicine (Tang *et al.* 2016; Qing *et al.* 2021). Based on gene homology analysis and some HPLC analytical methods, they conclude that LYD may not contain colchicine (Tang *et al.* 2016; Qing *et al.* 2021). Herein, we found that LYD leaves contained 3.08 ± 0.157 μg/g of colchicine. Elicitation with SA led to a 73.05% significant increment in the colchicine content of leaves. The positive impacts of SA application on alkaloid compound synthesis and accumulation in plants have been proven (Hadizadeh *et al.* 2019; Zavala‐gómez *et al.* 2021). Studies revealed that phenylalanine and tyrosine are the precursors for colchicine biosynthesized in several Liliaceae family herbaceous species (Nett, Lau and Sattely 2020; Nett and Sattely 2021; Stander, Papon and Courdavault 2021). Concordantly, functional analysis of up-regulated DEGs under SA treatment showed that they were mostly involved in tyrosine metabolism, phenylpropanoid biosynthesis and isoquinoline alkaloid biosynthesis. These results support the presence of colchicine in LYD leaves and suggest that the structural genes involved in this important nutraceutical synthesis in LYD may be different from those identified in other plants. Further genomics investigations on colchicine biosynthesis and regulation in *H. citrina* is required to clarify these statements. In addition, the integration of these findings shows that *H. citrina* may represent a source material for the biotechnological-assisted production of edible colchicine.

Besides, we identified 15 potential candidate genes, including polyphenol oxidase, transcription factors WRKY71-like, ethylene-responsive factors, peroxidase 12-likes, etc. These genes may be involved in vital developmental processes, environmental responses and colchicine metabolism in LYD. For instance, the roles of polyphenol oxidase, ERF and laccase in plant physiological metabolism and stress resistance have been documented (Shoji and Yuan 2021; Bai *et al.* 2023; Zhang 2023). Therefore, it is necessary to subject these potential candidate genes to functional characterization and verification studies in order to uncover their specific roles and deepen our knowledge of *H. citrina* biology, physiology and biochemistry. Functional genomics integrates molecular and cell biology studies to unravel a target gene’s structure, function and regulation (Kaushik, Kaushik and Sharma 2018). *CjWRKY1* was identified as the key transcriptional regulator of benzylisoquinoline alkaloid biosynthesis in Coptis japonica (Kato *et al.* 2007). Accordingly, the potential roles of the two WRKY71 candidate genes in colchicine biosynthesis in *H. citrina* have to be explored.

## Conclusions

In summary, this study found that yellow daylily foliar treatment with 2 mg/L SA significantly improved significantly growth, yield, and the leaf soluble sugar, soluble protein, ascorbic acid and colchicine contents by 15.15%, 19.54%, 30.45% and 73.05%, respectively. Notably, the leaf colchicine content was significantly increased from 3.08 ± 0.157 μg/g to 5.33 ± 0.462 μg/g. Through comparative transcriptomics analysis, 310 DEGs were identified, including 15 potential candidate genes. The plant hormone signal transduction, sulphur metabolism, phenylpropanoid biosynthesis, tyrosine metabolism, and isoquinoline alkaloid biosynthesis, were highly induced in SA-treated plants. Our results show the potential of SA application to improve yellow daylily production and quality. Moreover, they provide key knowledge for the biotechnological-assisted production of colchicine in *Hemerocallis spp*.

## Supporting Information

The following additional information is available in the online version of this article –

**Figure S1.** Volcano plot of differentially expressed genes (DEGs) between CK and T3.

Figure S2. GO annotation and enrichment results of DEGs.

Table S1. Summary of the high-quality RNA-seq data.

Table S2. List of all differentially expressed genes.

Table S3. List of primers used for the qRT-PCR analysis.

plae029_suppl_Supplementary_Materials

## Data Availability

The raw RNA-seq are available at https://www.ncbi.nlm.nih.gov/bioproject/?term=PRJNA1056505. The other data analyzed are included in this manuscript and its supplementary files.
