# Supplementary material for: Salicylic acid modulates secondary metabolism and enhanced colchicine accumulation in long yellow daylily (Hemerocallis citrina)
Source: AoB Plants. 2024 May 21;16(4):plae029. doi: 10.1093/aobpla/plae029 (PMC11232463; doi:10.1093/aobpla/plae029)
Supplement: plae029_suppl_Supplementary_Materials [file plae029_suppl_supplementary_materials.zip › aobplants-24039S1.pdf]

Salicylic acid modulates secondary metabolism and enhanced colchicine accumulation in long yellow daylily (*Hemerocallis citrina* Baroni)

Yeminzi Miao[1], Hanmei Li[2], Junjie Pan[1], Binxiong Zhou[1], Tianjun He[1], Yanxun Wu[3], Dayun Zhou[1], Weimin He[1*], Limin Chen[1*]

[1]Lishui Institute of Agricultural and Forestry Sciences, Lishui, Zhejiang 323000, China

[2]College of Forestry Science and Technology, Lishui Vocational & Technical College, Lishui, Zhejiang 323000, China

[3] Lishui Science & Technology Bureau, Lishui, Zhejiang 323000, China

***Correspondence:** zjjnming@163.com (W.H.) and clmit@zju.edu.cn (L.C.)

Abstract

Salicylic acid (SA) is an essential phytoregulator that is widely used to promote the synthesis of high-value nutraceuticals in plants. However, its application in daylily, an ornamental plant highly valued in traditional Chinese medicine, has not been reported. Herein, we investigated the exogenous SA-induced physiological, transcriptional, and biochemical changes in long yellow daylily (LYD). We found that 2 mg/L foliar SA treatment significantly improved LYD plant growth and yield. Transcriptome sequencing and differentially expressed genes analysis revealed that the phenylpropanoid biosynthesis, isoquinoline alkaloid biosynthesis, sulfur metabolism, plant hormone signal transduction, and tyrosine metabolism were significantly induced in SA-treated leaves. Many transcription factors and antioxidant system-related DEGs were induced under the SA treatment. Biochemical analyses showed that the leaf contents of soluble sugar (SS), soluble protein (Cpr), ascorbic acid (AsA), and colchicine were significantly increased by 15.15% (from 30.16±1.301 to 34.73±0.861 mg/g), 19.54% (from 60.3±2.227 to 72.08±1.617 mg/g), 30.45% (from 190.1±4.56 to 247.98±11.652 µg/g), and 73.05% (from 3.08±0.157 to 5.33±0.462 µg/g), respectively, under the SA treatment. Furthermore, we identified 15 potential candidate genes for enhancing the growth, production, and phytochemical content of LYD. Our results provide support for the bioaccumulation of colchicine in yellow daylily and valuable resources for biotechnological-assisted production of this important nutraceutical in *Hemerocallis spp*.

Keywords: Colchicine, *Hemerocallis citrina*, Salicylic acid, Phytoregulator, Transcriptomics, Candidate gene.

1. Introduction

Long yellow daylily (LYD, *Hemerocallis citrina* Baroni) is a monocotyledonous perennial herbaceous plant from the family Liliaceae (Qing *et al.* 2021). It is an important vegetable and ornamental plant with tremendous applications as a functional food, flavoring agent, and raw material in traditional Chinese medicine (Guo *et al.* 2022, 2023). LYD is widely distributed in Mongolia, Russia, China, Japan, Korea, and Europe and is popular for its attractive flowers (Guo *et al.* 2022, 2023). It is a material of choice in treating depression and has also shown antioxidant, anti-inflammatory, anti-constipation, anti-lactation deficiency, and neuroprotective abilities (Tian *et al.* 2017; Matraszek-Gawron *et al.* 2019; Zhong *et al.* 2021; Ma *et al.* 2022, 2023; Jiang *et al.* 2023; Liang *et al.* 2023). It is a rich source of nutrients (amino acids, carbohydrates, phosphorus, calcium, iron, and vitamins C, B1, B2, and B5) and functional substances, such as lecithin, alkaloids, phenolic acids, flavonoids, saponins (Guo *et al.* 2022; Li *et al.* 2022). With its important ornamental and medicinal potentials, enhancing the production and nutraceutical content of LYD will contribute to its sustainable use and value addition.

Colchicine (an alkaloid) is one of the most ancient remedies used in diverse medical disciplines, such as dermatology, immunology, oncology, cardiology, nephrology, etc. (Solak *et al.* 2017; Dasgeb *et al.* 2018; Robinson and Chan 2018; Siak *et al.* 2021; Huber *et al.* 2023). Since ancient times, colchicine has been the most effective drug for the treatment of neutrophilic inflammation, mainly gout, amyloidosis, and familial Mediterranean fever (Richette and Bardin 2010; Dalbeth, Lauterio and Wolfe 2014; Leung, Yao Hui and Kraus 2015; Dasgeb *et al.* 2018; Schattner 2022). It is also effective in treating cancers (Kumar, Sharma and Mondhe 2016), acute pericarditis (Imazio *et al.* 2013; Siak *et al.* 2021; Huet *et al.* 2022), atherosclerosis (Meyer-Lindemann *et al.* 2022), and coronary diseases (COVID-19) (Nidorf *et al.* 2020; Mikolajewska *et al.* 2021). Besides its high medicinal value, colchicine is an important compound used in biotechnology to induce plant diploidization and polyploidization (Ślusarkiewicz-Jarzina *et al.* 2017; Tammu, Nuringtyas and Daryono 2021; Wu *et al.* 2022; Bajpai and Chaturvedi 2023). As for many plant-derived nutraceuticals, colchicine production still relies on natural resources from which it is extracted. Studies revealed that it is biosynthesized in several herbaceous species members of the Liliaceae family from phenylalanine and tyrosine (Nett, Lau and Sattely 2020; Nett and Sattely 2021; Stander, Papon and Courdavault 2021). Unfortunately, it is produced in very low quantities in whole plants. Therefore, enhancing its availability in source materials is

of great interest. The report by Traub et al. indicated that *Hemerocallis* species may contain an appreciable amount of colchicine (Traub 1949). We thus speculated that *H. citrina* could represent a source of edible colchicine production for medical and biotechnological applications.

SA is a vital plant regulator that affects diverse growth and development processes, including seed germination, pigment accumulation, stomatal movements, photosynthesis, heat production, ethylene biosynthesis, enzyme activities, nutrient uptake, abscission reversal, membrane functions, flower induction, and metabolic activities (Larqué-Saavedra 2007; Ali 2021). Owing to its hormone activity, SA has been widely applied to enhance plants' abiotic and biotic stress tolerance and to promote secondary metabolite biosynthesis and accumulation (Larqué-Saavedra 2007; Ali 2021; Li, Sun and Liu 2022; Liu *et al.* 2022; Monteiro *et al.* 2022). It has been applied to improve the content of anthocyanins and polyphenols in grapevine (Oraei *et al.* 2019; Blanch, Gómez-Jiménez and del Castillo 2020); induce flavonoid synthesis in wheat (Gondor *et al.* 2016); stimulate alkaloid accumulation in *Arthrospira platensis* (Hadizadeh *et al.* 2019); promote glucosinolates accumulation in *Brassica oleracea* (Yi *et al.* 2016); and improve the quality of blueberry fruits (Jiang *et al.* 2022b, 2022a). Hence, we hypothesized that exogenous application of SA may improve LYD growth and production, and enhance the synthesis and accumulation of nutrients, colchicine and other secondary metabolites. Understanding the physiological, biochemical, and molecular changes associated with SA applications in LYD, will offer fundamental resources for improving the crop's performance and quality.

The main objective of the present study was to reveal the physiological, biochemical, and transcriptional changes associated with exogenous SA treatment of LYD. We determined the optimal concentration of SA to improve LYD growth, development, and yield. We investigated the impact of the optimal SA concentration on nutrients (soluble sugar and soluble protein), antioxidant enzymes, flavone, total phenolic, ascorbic acid, and colchicine contents of LYD leaves. Furthermore, we performed a comparative transcriptomics analysis and unveiled significantly induced pathways and potential candidate genes. Our findings provide fundamental resources for biotechnological-assisted edible colchicine production in LYD.

2. Materials and Methods

2.1. Plant material and experimental procedures

LYD seedlings preserved by the Lishui Institute of Agricultural and Forestry Sciences were used in this study. The roots were transplanted into a nutrient bowl (height 17 cm, diameter 20 cm) and cultivated in an incubator (temperature 28 ± 2 ℃, 14 h light/10 h dark, humidity 50-60%). When the seedlings reached 8-10 cm in height, they were transplanted into pots (one individual plant per pot) in an insect-free greenhouse and allowed to grow for 20 days until the 5-leaf stage. Uniform seedlings with healthy plants were selected and divided for further experimentation. The day/night temperatures and relative humidity in the greenhouse were 24 ± 1 ℃/20 ± 1 ℃ and 60 ± 5%, respectively. The diameter and height of the pot were 20 cm and 30 cm, respectively. Six treatments, including CK (control), T1 (0.5 mg/L), T2 (1 mg/L), T3 (2 mg/L), T4 (4 mg/L), and T5 (6 mg/L), with six replications were set. All plants were watered normally. SA was dissolved in dimethyl sulfoxide and diluted as needed to the desired concentration. 1 ml Tween-20 was added to each 1 L of aqueous solution and sprayed on the leaves. After 14 days following the foliar SA treatment, physiological indicators such as plant height, leaf length, and leaf width were measured, and samples (the middle part of the three leaves) were collected for biochemical indicators and transcriptomics analyses. The total chlorophyll content was assessed on three fully opened leaves using a SPAD meter with three technical measures per leaf. After the yellow cauliflower flower buds grew, commercial flower buds were collected for yield-related traits measurement. Samples for biochemical traits and transcriptomics analysis were directly frozen in liquid nitrogen and kept at -80ºC until used.

2.2.Evaluation of biochemical indicators

Based on the results of growth and yield indicators, only the CK and T3 groups were selected for biochemical and transcriptome sequencing. Eight biochemical traits, including antioxidant enzymes (SOD and CAT), soluble sugar (SS), soluble protein (Cpr), total phenols, total flavone, reduced ascorbic acid (AsA), and colchicine were selected to explore SA-induced metabolic changes. All biochemical tests were performed with three biological and technical replications.

For the evaluation of Cpr, SS, and the activity of CAT (catalase) and SOD (superoxide dismutase), 100 mg of each sample were ground in a pre-cooled mortar with liquid nitrogen and extracted with 1.5 mL phosphate buffer (1 mM EDTA, 10 mM cysteine, pH 7.5). Then, all extracts were collected separately and centrifuged (10,000 g for 15 min). The Cpr was evaluated

using the BCA (bicinchoninic acid assay) method. In brief, 4 μL of the extract and 200 μL of BCA working solution were mixed. After incubation at 60 °C for 30 min, the absorbance was recorded at 562 nm using a microplate reader (SpectraMax ABS plus, Molecular Devices, CA, USA). The extraction buffer was used as a control, and the bovine serum albumin was used as standard (y = 4.2274x -0.3374, R^2 = 0.9962). The results were expressed per mg of fresh weight (mg/g FW). The SS assay kit was used for soluble sugar content assessment (Buysse and Merckx 1993). Similarly, the activities of CAT and SOD were measured using their respective specific kits (Yan *et al.* 2023).

For the evaluation of total phenols content, total flavonoid content, and reduced ascorbic acid (AsA) content, 100 mg frozen samples were extracted with 10 mL of ethanol and water (80: 20 v/v) at 37 ℃ for 2 hours. Next, the extracts were centrifuged at 5,000 g for 20 min. The total flavonoids was determined using the NaNO2-AlCl3-NaOH method (Luan *et al.* 2023). The total Phenols was evaluated using the Folin Phenol biochemical kit (Sun *et al.* 2023a). The AsA content was evaluated using the red phenanthroline colorimetric method (plant ascorbic acid content detection kit) (Sun *et al.* 2023b). All the test kits were obtained from Norminkoda Biotechnology Co., Ltd. Wuhan, China.

2.3. Evaluation of colchicine content

Colchicine extraction and quantification were performed following the method described by Al-Fayyad et al. (Al-Fayyad *et al.* 2002). The samples were dried and ground to powder. Then 15 g was extracted with methanol–water (1: 8) at 30 ºC for 24 h, repeated five times. The crude extracts were pooled, filtered, and centrifugated (12,000 g for 15 min). Next, the supernatants were evaporated to dryness at 55 ºC. The residues were dissolved in 5% acetic acid, followed by extraction with petroleum ether to remove non-alkaloid compounds. The aqueous acid residues were further extracted with ethyl ether, followed by a pH adjustment to 9 with ammonium hydroxide. Finally, the aqueous extracts were extracted with chloroform, followed by the evaporation of the chloroform to dryness. The residues were weighted, and the extracts were reconstituted with methanol–water for HPLC (high-performance liquid chromatography) analysis using an Agilent 1260. The HPLC conditions were as follows: Column, PL1512-5501 ChromSphe C18, 250 mm× 4.6 mm, 5 μm; Flow rate, 0.5 mL/min; Injection volume, 10 μL;

Mobile phase, methanol-water 50:50; Detector wavelength, 350 nm. Pure colchicine (C9754) was purchased from Sigma-Aldrich (St. Louis, MO, USA) for calibration.

2.4. RNA Extraction, library construction, sequencing, and alignment

Total RNA from leaf samples was extracted with a Trizol reagent kit (Invitrogen, Carlsbad, CA, USA) as per the manufacturer's specifications. The genomic DNA was discarded using DNase I (TaKara, Beijing, China). RNA quality was investigated on an Agilent 2100 Bioanalyzer (Agilent Technologies, Palo Alto, CA, USA) and quantified using the ND-2000 (NanoDrop Technologies). Only high-quality RNA (OD260/280 = 1.8~2.2, OD260/230 ≥ 2.0, RIN ≥ 6.5, 28S:18S≥1.0, >1 μ g) samples were used for sequencing library construction using TruSeqTM RNA sample preparation Kit (Illumina, San Diego, CA). After qualified mRNA fragmentation, cDNAs were constructed using NEB (Next Ultra RNA Library Prep Kit, Ipswich, MA, USA), and adapters were ligated. The resulting cDNA library was sequenced on the Illumina sequencing platform (HiSeq xten/NovaSeq6000 sequencer). SeqPrep (https://github.com/jstjohn/SeqPrep) and Sickle (https://github.com/najoshi/sickle) software were used to check the quality of raw paired-end. The clean reads were aligned to the *H. citrina* reference genome (Qing *et al.* 2021) by the HISAT2 (http://ccb.jhu.edu/software/hisat2/index.shtml) software (Kim, Langmead and Salzberg 2015). Finally, we assembled the mapped reads using StringTie (http://www.string-db.org/) (Pertea *et al.* 2015).

2.5. Differentially expressed genes (DEGs) and functional enrichment analysis

The expression level of transcripts was computed according to the transcripts per million reads (TPM) method, and RSEM (http://deweylab.biostat.wisc.edu/rsem/) was used to quantify each gene abundance (Li and Dewey 2011). DEGs analysis was carried out using the DESeq2 software (Love, Huber and Anders 2014) at FDR (false discovery rate) < 0.05 and |fold change| ≥ 1. KEGG (Kyoto Encyclopedia of Genes and Genomes, http://www.genome.jp/kegg/kaas) and GO (Genes Ontology, http://geneontology.org/) enrichment analyses were achieved using KOBAS (2.0) and GO seq software, respectively. Significant enrichment terms were screened out at *P*-value ＜0.05.

2.6. Quantitative RT–PCR analysis

The RNA was extracted from leaf samples using a modified CTAB method (Kanani *et al.* 2019). Reverse transcription (RT) was conducted with the Monad 1st Strand cDNA Synthesis Kit, and the qRT-PCR analysis was achieved using Tb Green® Premix Ex Taq™ II (Takara, Beijing, China) as previously described (Huang *et al.* 2022, 2023). All samples had three biological and technical replicates. Actin gene (*J01298*) was used as an internal control (Hou *et al.* 2017) to normalize the expression levels of target genes via the $2^{-\Delta\Delta CT}$ method (Livak and Schmittgen 2001). The NCBI's primer designing tool, PRIMER-BLAST software was used to design specific primers for each gene (**Table S3**).

2.7. Data analysis

Excel 2016 software,GraphPad Prism v9 (GraphPad Software Inc., La Jolla, CA, USA), SR plots (Tang *et al.* 2023), and R (version 4.3) were used for data processing and graph construction. PCA (principal component analysis) analysis was carried out using the R package prcomp. ANOVA (analysis of variance) and post hoc test (Tukey test) were performed for multiple comparisons at $P < 0.05$. For statistical differences between CK and T3, a t-test was performed and the significance was set at $P < 0.05$. Heatmaps were constructed using TBtools-II software (Chen *et al.* 2023).

3. Results

3.1. Impact of exogenous salicylic acid on *H. citrina* growth and yield

To explore the impacts of exogenous SA application on the growth and production of *H. citrina* plants, we proceeded to morphological observations and investigated the variation in agronomic traits under different SA treatments, including T1 (0.5 mg/L), T2 (1 mg/L), T3 (2 mg/L), T4 (4 mg/L), and T5 (6 mg/L). The morphologies of the plants fourteen days after SA treatment are shown in **Figures 1A-F**. In general, low SA concentrations (0.5 - 2 mg/L) improved the growth and yield parameters, whereas high SA concentration (> 4 mg/L) showed opposite effects (**Figure 1**). The growth and yield traits were significantly improved under T3 than under other treatments (**Figure 1**). For instance, the plant height, leaf length, leaf width, alabastrum length, alabastrum wide, alabastrum weight, and yield were significantly increased

under T3 compared to the control (CK) and other treatments (**Figures 1G-I, K-N**). No major differences were observed in the chlorophyll content (**Figure 1J**). These results showed that T3 was the optimal SA concentration to promote growth and higher yield in *H. citrina*. Therefore, we selected T3 for further analyses.

3.2. Salicylic acid-induced higher accumulation of colchicine, ascorbic acid, soluble protein, and soluble sugar

To reveal the SA-induced biochemical changes in *H. citrina*, we evaluated the activity of antioxidant enzymes (CAT and SOD) and the content of soluble protein (Cpr), soluble sugar, reduced ascorbic acid (AsA), total phenol, flavone, and colchicine in CK and T3 leaves (**Figure 2**). No significant difference in CAT activity between CK and T3 was recorded, whereas the activity of SOD in CK was significantly higher than that in T3 (**Figures 2A, B**). The total phenol and flavone contents of CK and T3 were also statistically identical (**Figures 2C, D**). Regarding the other biochemical traits, the SA application induced a significant increase in the content of Cpr, soluble sugar, AsA, and colchicine in T3 compared to CK (**Figures 2E-H**). For instance, the soluble sugar, Cpr, AsA, and colchicine content in *H. citrina* leaves were increased by 15.15, 19.54, 30.45, and 73.05 %, respectively. Of them, colchicine content was the most significantly increased, with an improvement from 3.08±0.157 μg/g (CK) to 5.33±0.462 μg/g (T3) (**Figure 2H**). The leaf SS, Cpr, and AsA contents significantly increased from 30.16±1.301 to 34.73±0.861 mg/g, 60.3±2.227 to 72.08±1.617 mg/g, and 190.1±4.56 to 247.98±11.652 μg/g, respectively (**Figures 2E-G**).

3.3. Comparative transcriptome sequencing and differentially expressed genes (DEGs)

To insights into the SA-induced molecular changes in *H. citrina* , CK and T3 leaf samples were subjected to transcriptome sequencing. The RNA sequencing yielded 38,467,202 to 88,292,204 bp of raw reads, with clean reads ranging from 37,765,600 to 88,088,068 bp (**Table S1**). The Q20, Q30, and GC content varied from 96.46 to 97.59 %, 90.09 to 92.98 %, and 45.8 to 47.91%, respectively (**Table S1**), indicating the high quality of the RNA-seq data. The total mapping rates against the reference genome were 79.81 – 85.95 %, respectively (**Table S1**). Principal component analysis of samples showed that the transcriptomes of CK and T3 were different and could be discriminated by PC1 of 94.8 % (**Figure 3A**).

To examine transcriptional changes under T3 treatment, we screened out all DEGs. A total of 310 DEGs were identified, including 200 significantly induced in T3 (**Figure 3B** and **Table S2**). The volcano plot of DEGs is presented in **Figure S1**. To unveil the key molecular mechanisms affected by the SA treatment, we carried out GO and KEGG annotation and enrichment analyses of DEGs. The general GO result is presented in **Figure S2**. The most induced GO terms related to biological processes were flavonoid metabolic process, protein-chromophore linkage, response to Karrikin, and sulfate metabolism (**Figure S2A**). Regarding cellular components, intrinsic and integral components of membrane, membrane part, extracellular regions, and external encapsulating structure were the main enriched (**Figure S2B**). Meanwhile, in the molecular function, the DEGs were mostly assigned to cofactor binding, cation binding, metal ion binding, and oxidoreductase activity (**Figure S2C**). KEGG enrichment revealed that the DEGs were primarily involved in photosynthesis, circadian rhythm-plant, flavonoid biosynthesis, phenylpropanoid biosynthesis, sulfur metabolism, plant hormone signal transduction, and isoquinoline alkaloid biosynthesis (**Figure 4A**).

3.4. Influence of salicylic acid on antioxidant system and phenylpropanoid biosynthesis

The KEGG analysis indicated that the SA application modulated the antioxidant system and phenylpropanoid metabolism. Therefore, we explored the expression patterns of DEGs related to the antioxidant system, flavonoid biosynthesis, phenylpropanoid biosynthesis, and tyrosine metabolism (**Figures 4B, C and 5A**). Most of the DEGs related to the antioxidant system, including peroxidases, glutathione S-transferase, and ascorbate oxidase were up-regulated under T3 (**Figure 4B**). Five out of six DEGs related to phenylpropanoid biosynthesis/isoquinoline alkaloid biosynthesis, including laccase 2 (*gene-HHC001507*), polyphenol oxidase (*gene-HHC030644*), etc., were induced under T3 (**Figure 4C**). Regarding flavonoid biosynthesis, 18 DEGs were screened out, including eight up-regulated and 10 down-regulated under the SA treatment (**Figure 5A**).

3.5. Transcription factors (TFs) and phytohormone-related DEGs

TFs and phytohormones are critical for plant growth and developmental processes. We identified 16 and two TF and phytohormone-related DEGs, respectively, and examined their expression patterns under CK and T3 (**Figure 5B**). Thirteen of the TF-related DEGs, including

one NAC (*gene-HHC015099*), three WRKYs (*gene-HHC027357*, *gene-HHC034893*, and *gene-HHC042262*), three MYBs (*gene-HHC014072, gene-HHC005082* and *gene-HHC052364*), two ERFs (*gene-HHC030807* and *gene-HHC036301*), two TIFYs (*gene-HHC040139* and *new_gene475*), one ZAT (*gene-HHC036739*), and one DIVARICATA (*gene-HHC042716*) were induced under the SA treatment (**Figure 5B**). Regarding the phytohormone-related DEGs, only *gene-HHC044569*, encoding a jasmonic acid-amido synthetase was up-regulated under T3 (**Figure 5B**).

3.6. Potential candidate genes and qRT-PCR validation

Candidate genes are essential for the molecular-assisted breeding and biotechnology perspectives. To identify potential candidate genes for targeted improvement of *H. citrina* plant growth, yield, and content of nutraceuticals, we focused on pathways that were significantly induced under T3. We screened out a total of 15 potential candidate genes, including one polyphenol oxidase (*gene-HHC030644*), two transcription factors WRKY71-like (*gene-HHC042262* and *gene-HHC034893*), two ethylene-responsive factors (*gene-HHC030807* and *gene-HHC036301*), two peroxidase 12-likes (*gene-HHC011956* and *gene-HHC048544*), etc. (**Table 1**). Of them, *new_gene521* (a new gene with unknown function) was the most significantly induced under T3 (**Table 1**).

To validate the RNA-seq data and confirm the potential candidate genes, they were subjected to qRT-PCR analysis. As shown in **Figure 6**, the qRT-PCR results confirmed that these genes were significantly up-regulated under T3 compared to CK.

4. Discussion

Improving plant growth and content of higher-medicinal values functional compounds is essential to sustain their use in human health. Thus, the present study showed the potential of foliar SA treatment to enhance the growth, yield, ascorbic acid, and colchicine content of *H. citrina*. Moreover, the involved molecular mechanisms were revealed through comparative transcriptomics analysis.

SA is a critical signal molecule that modulates plant immunity and growth processes through interplay with other phytohormones to regulate cell division, expansion, and metabolism (Li, Sun and Liu 2022). It has been applied at low concentrations to enhance the growth,

development, and productivity of many horticultural plants, such as *Capsicum chinense* and *Brosimum alicastrum* (Tucuch-Haas *et al.* 2017). Research on wheat and maize has also demonstrated the positive effects of exogenous SA on plant growth, development, and productivity under diverse environmental conditions (El-Mergawi and Abd El-Wahed 2020; Mohammed *et al.* 2023). As per previous reports in other plants, we found that low SA concentrations (0.5 - 2 mg/L) improved the growth and yield parameters of *H. citrina*, whereas high SA concentration (> 4 mg/L) showed opposite effects. Particularly, the plant height, leaf length, leaf width, alabastrum length, alabastrum wide, alabastrum weight, and yield were significantly increased under 2 mg/L (T3) SA treatment compared to the control (CK) and other treatments. Moreover, we recorded a significant increase in the content of Cpr, soluble sugar, AsA, and colchicine under T3 compared to CK. These results indicate that 2 mg/L is the optimal SA concentration to induce signaling mechanisms toward growth, production, and quality improvement in *H. citrina*. Concordantly, functional analysis of DEGs revealed that sulfur metabolism and plant hormone signal transduction (TFs and phytohormone-related DEGs) were significantly induced under T3 treatment. Sulfur metabolism is critical for all organisms, as sulfur is required for the biosynthesis of antioxidants, sulfolipids, amino acids (cysteine and methionine), secondary metabolites, proteins, SAM (S-adenosylmethionine, precursor of ethylene), and cofactors (De Kok *et al.* 2012). The antioxidant system-related DEGs were up-regulated under T3. These findings suggest that 2 mg/L SA application might improve *H. citrina* plant tolerance capability to stresses. Taken together, the above results infer that 2 mg/L foliar SA application could be recommended to promote growth, production, and quality improvement in *H. citrina*.

Colchicine is a pharmacologically active tricyclic alkaloid used for various medical applications (Solak *et al.* 2017; Dasgeb *et al.* 2018; Robinson and Chan 2018; Siak *et al.* 2021; Huber *et al.* 2023). However, an uncontrolled increase in colchicine concentration can lead to toxicity and death due to overdose (Krishna and Shivankar 2021). The presence of colchicine in LYD has been associated with poisoning issues (Traub 1949). Accordingly, Tang et al. and Qing et al. have performed a series of analyses regarding LYD containing colchicine (Tang *et al.* 2016; Qing *et al.* 2021). Based on gene homology analysis and some HPLC analytical methods, they conclude that LYD may not contain colchicine (Tang *et al.* 2016; Qing *et al.* 2021). Herein, we found that LYD leaves contained 3.08±0.157 μg/g of colchicine. Elicitation with SA led to a

73.05% significant increment in the colchicine content of leaves. The positive impacts of SA application on alkaloid compound synthesis and accumulation in plants have been proven (Hadizadeh *et al.* 2019; Zavala - g6mez *et al.* 2021). Studies revealed that phenylalanine and tyrosine are the precursors for colchicine biosynthesized in several Liliaceae family herbaceous species (Nett, Lau and Sattely 2020; Nett and Sattely 2021; Stander, Papon and Courdavault 2021). Concordantly, functional analysis of up-regulated DEGs under SA treatment showed that they were mostly involved in tyrosine metabolism, phenylpropanoid biosynthesis, and isoquinoline alkaloid biosynthesis. These results support the presence of colchicine in LYD leaves and suggest that the structural genes involved in this important nutraceutical synthesis in LYD may be different from those identified in other plants. Further genomics investigations on colchicine biosynthesis and regulation in *H. citrina* is required to clarify these statements. In addition, the integration of these findings shows that *H. citrina* may represent a source material for the biotechnological-assisted production of edible colchicine.

Besides, we identified 15 potential candidate genes, including polyphenol oxidase, transcription factors WRKY71-like, ethylene-responsive factors, peroxidase 12-likes, etc. These genes may be involved in vital developmental processes, environmental responses, and colchicine metabolism in LYD. For instance, the roles of polyphenol oxidase, ERF, and laccase in plant physiological metabolism and stress resistance have been documented (Shoji and Yuan 2021; Bai *et al.* 2023; Zhang 2023). Therefore, it is necessary to subject these potential candidate genes to functional characterization and verification studies in order to uncover their specific roles and deepen our knowledge of *H. citrina* biology, physiology, and biochemistry. Functional genomics integrates molecular and cell biology studies to unravel a target gene's structure, function, and regulation (Kaushik, Kaushik and Sharma 2018). *CjWRKY1* was identified as the key transcriptional regulator of benzylisoquinoline alkaloid biosynthesis in Coptis japonica (Kato *et al.* 2007). Accordingly, the potential roles of the two WRKY71 candidate genes in colchicine biosynthesis in *H. citrina* have to be explored.

5. Conclusions

In summary, this study found that yellow daylily foliar treatment with 2 mg/L SA significantly improved significantly growth, yield, and the leaf soluble sugar, soluble protein, ascorbic acid, and colchicine contents by 15.15%, 19.54%, 30.45%, and 73.05%, respectively.

Notably, the leaf colchicine content was significantly increased from 3.08±0.157 μg/g to 5.33±0.462 μg/g. Through comparative transcriptomics analysis, 310 DEGs were identified, including 15 potential candidate genes. The plant hormone signal transduction, sulfur metabolism, phenylpropanoid biosynthesis, tyrosine metabolism, and isoquinoline alkaloid biosynthesis, were highly induced in SA-treated plants. Our results show the potential of SA application to improve yellow daylily production and quality. Moreover, they provide key knowledge for the biotechnological-assisted production of colchicine in *Hemerocallis spp*.

Supplementary Information

The supplementary files are available in the online version of the article.

Figure S1. Volcano plot of differentially expressed genes (DEGs) between CK and T3**. Figure S2**: GO annotation and enrichment results of DEGs; **Table S1**: Summary of the high-quality RNA-seq data; **Table S2**: List of all differentially expressed genes; **Table S3**: List of primers used for the qRT-PCR analysis.

Data availability

The raw RNA-seq are available at https://www.ncbi.nlm.nih.gov/bioproject/?term=PRJNA1056505. The other data analyzed are included in this manuscript and its supplementary files.

Declaration of competing interests

All authors declare that they have no personal, financial, or other conflicts of interest.

Funding

This research was funded by the Lishui Science and Technology Plan Project (2021GYX08).

CRedit authorship contribution statement

Y.M., methodology, data curation, software, and writing—original draft preparation; H.L., J.P., and B.Z., formal analysis, validation, and software; T.H., Y.W., and D.Z., investigation, resources, and visualization; W.H. and L.C., conceptualization, writing—review and editing, and supervision; L.C., project administration and funding acquisition. All authors read and approved the final manuscript.

Acknowledgments

Not applicable.

References

Al-Fayyad M, Alali F, Alkofahi A *et al.* Determination of colchicine content in Colchicum hierosolymitanum and Colchicum tunicatum under cultivation. *Nat Prod Lett* 2002;**16**:395–400.

Ali B. Salicylic acid: An efficient elicitor of secondary metabolite production in plants. *Biocatal Agric Biotechnol* 2021;**31**:1–10.

Bai Y, Ali S, Liu S *et al.* Characterization of plant laccase genes and their functions. *Gene* 2023;**852**:147060.

Bajpai R, Chaturvedi R. In vitro production of doubled haploid plants in Camellia spp. and assessment of homozygosity using microsatellite markers. *J Biotechnol* 2023;**361**:89–98.

Blanch GP, Gómez-Jiménez MC, del Castillo MLR. Exogenous Salicylic Acid Improves Phenolic Content and Antioxidant Activity in Table Grapes. *Plant Foods Hum Nutr* 2020;**75**:177–83.

Buysse J, Merckx R. An Improved Colorimetric Method to Quantify Sugar Content of Plant Tissue. *J Exp Bot* 1993;**44**:1627–9.

Chen C, Wu Y, Li J *et al.* TBtools-II: A “one for all, all for one” bioinformatics platform for biological big-data mining. *Mol Plant* 2023;**16**:1733–42.

Dalbeth N, Lauterio TJ, Wolfe HR. Mechanism of action of colchicine in the treatment of gout. *Clin Ther* 2014;**36**:1465–79.

Dasgeb B, Kornreich D, McGuinn K *et al.* Colchicine: an ancient drug with novel applications. *Br J Dermatol* 2018;**178**:350–6.

El-Mergawi RA, Abd El-Wahed MSA. Effect of exogenous salicylic acid or indole acetic acid on their endogenous levels, germination, and growth in maize. *Bull Natl Res Cent* 2020;**44**, DOI: 10.1186/s42269-020-00416-7.

Gondor OK, Janda T, Soós V *et al.* Salicylic acid induction of flavonoid biosynthesis pathways

in wheat varies by treatment. *Front Plant Sci* 2016;**7**:1–12.

Guo A, Li S, Yang Y *et al.* Lecithin extraction optimisation and synthesis in Hemerocallis citrina Baroni. *Sci Hortic (Amsterdam)* 2022;**293**:110682.

Guo A, Yang Y, Wu J *et al.* Lipidomic and transcriptomic profiles of glycerophospholipid metabolism during Hemerocallis citrina Baroni flowering. *BMC Plant Biol* 2023;**23**:1–15.

Hadizadeh M, Ofoghi H, Kianirad M *et al.* Elicitation of pharmaceutical alkaloids biosynthesis by salicylic acid in marine microalgae Arthrospira platensis. *Algal Res* 2019;**42**:101597.

Hou F, Li S, Wang J *et al.* Identification and validation of reference genes for quantitative real-time PCR studies in long yellow daylily, Hemerocallis citrina Borani. *PLoS One* 2017;**12**:1–14.

Huang X, Chu G, Wang J *et al.* Integrated metabolomic and transcriptomic analysis of specialized metabolites and isoflavonoid biosynthesis in Sophora alopecuroides L. under different degrees of drought stress. *Ind Crops Prod* 2023;**197**:116595.

Huang Y, Xun H, Yi G *et al.* Integrated Metabolomic and Transcriptomic Analysis Reveals the Effect of Artificial Shading on Reducing the Bitter Taste of Bamboo Shoots. *Horticulturae* 2022;**8**, DOI: 10.3390/horticulturae8070594.

Huber SM, Navarini A, Brandt O *et al.* Colchicine - Renaissance of an "ancient" drug. *J der Dtsch Dermatologischen Gesellschaft = J Ger Soc Dermatology JDDG* 2023;**21**:239–43.

Huet F, Delbaere Q, Fauconnier J *et al.* Colchicine: protection of the brain beyond the heart? *Expert Rev Clin Immunol* 2022;**18**:101–3.

Imazio M, Brucato A, Cemin R *et al.* A Randomized Trial of Colchicine for Acute Pericarditis. *N Engl J Med* 2013;**369**:1522–8.

Jiang B, Fang X, Fu D *et al.* Exogenous salicylic acid regulates organic acids metabolism in postharvest blueberry fruit. *Front Plant Sci* 2022a;**13**:1–11.

Jiang B, Liu R, Fang X *et al.* Effects of salicylic acid treatment on fruit quality and wax composition of blueberry (Vaccinium virgatum Ait). *Food Chem* 2022b;**368**:130757.

Jiang N, Zhang Y, Yao C *et al.* Hemerocallis citrina Baroni ameliorates chronic sleep

deprivation-induced cognitive deficits and depressive-like behaviours in mice. *Life Sci Sp Res* 2023, DOI: 10.1016/j.lssr.2023.04.001.

Kanani P, Shukla YM, Modi AR *et al.* Standardization of an efficient protocol for isolation of RNA from Cuminum cyminum. *J King Saud Univ - Sci* 2019;**31**:1202–7.

Kato N, Dubouzet E, Kokabu Y *et al.* Identification of a WRKY protein as a transcriptional regulator of benzylisoquinoline alkaloid biosynthesis in Coptis japonica. *Plant Cell Physiol* 2007;**48**:8–18.

Kaushik S, Kaushik S, Sharma D. Functional genomics. *Encycl Bioinforma Comput Biol ABC Bioinforma* 2018;**1**–**3**:118–33.

Kim D, Langmead B, Salzberg SL. HISAT: a fast spliced aligner with low memory requirements. *Nat Methods* 2015;**12**:357–60.

De Kok LJ, Tabe L, Tausz M *et al.* eds. *Sulfur Metabolism in Plants*. Dordrecht: Springer Netherlands, 2012.

Krishna R, Shivankar B. A review on liquid chromatographic analysis of colchicine in the forensic and medical perspective. *Toxicol Anal Clin* 2021;**33**:276–87.

Kumar A, Sharma PR, Mondhe DM. Potential anticancer role of colchicine-based derivatives: An overview. *Anticancer Drugs* 2016;**28**:250–62.

Larqué-Saavedra A and RM-M. Effects of salicylic acid on the bioproductivity of plants. Salicylic acid: a plant hormone. *Dordr Springer* 2007:15–23.

Leung YY, Yao Hui LL, Kraus VB. Colchicine-Update on mechanisms of action and therapeutic uses. *Semin Arthritis Rheum* 2015;**45**:341–50.

Li A, Sun X, Liu L. Action of Salicylic Acid on Plant Growth. *Front Plant Sci* 2022;**13**, DOI: 10.3389/fpls.2022.878076.

Li B, Dewey CN. RSEM: accurate transcript quantification from RNA-Seq data with or without a reference genome. *BMC Bioinformatics* 2011;**12**:323.

Li M, Liu H, Xiao J *et al.* Research Progress on Bioactive Components, Biological Activities, and Processing Technology of Daylily (Hemerocallis citrina Baroni). *Sci Technol Food Ind*

2022;**43**:427–35.

Liang Y, Wei X, Ren R *et al.* Study on Anti-Constipation Effects of Hemerocallis citrina Baroni through a Novel Strategy of Network Pharmacology Screening. *Int J Mol Sci* 2023;**24**, DOI: 10.3390/ijms24054844.

Liu J, Qiu G, Liu C *et al.* Salicylic Acid, a Multifaceted Hormone, Combats Abiotic Stresses in Plants. *Life* 2022;**12**, DOI: 10.3390/life12060886.

Livak KJ, Schmittgen TD. Analysis of Relative Gene Expression Data Using Real-Time Quantitative PCR and the 2−ΔΔCT Method. *Methods* 2001;**25**:402–8.

Love MI, Huber W, Anders S. Moderated estimation of fold change and dispersion for RNA-seq data with DESeq2. *Genome Biol* 2014;**15**:550.

Luan A, Zhang W, Yang M *et al.* Unveiling the molecular mechanism involving anthocyanins in pineapple peel discoloration during fruit maturation. *Food Chem* 2023;**412**:135482.

Ma T, Sun Y, Lin J *et al.* Chemical constituents and mechanisms from Hemerocallis citrina Baroni with anti-neuroinflammatory activity. *J Funct Foods* 2023;**102**:105427.

Ma T, Sun Y, Wang L *et al.* An Investigation of the Anti-Depressive Properties of Phenylpropanoids and Flavonoids in Hemerocallis citrina Baroni. *Molecules* 2022;**27**, DOI: 10.3390/molecules27185809.

Matraszek-Gawron R, Chwil M, Terlecka P *et al.* Recent studies on anti-depressant bioactive substances in selected species from the genera hemerocallis and gladiolus: A systematic review. *Pharmaceuticals* 2019;**12**, DOI: 10.3390/ph12040172.

Meyer-Lindemann U, Mauersberger C, Schmidt AC *et al.* Colchicine Impacts Leukocyte Trafficking in Atherosclerosis and Reduces Vascular Inflammation. *Front Immunol* 2022;**13**:1–12.

Mikolajewska A, Fischer AL, Piechotta V *et al.* Colchicine for the treatment of COVID-19. *Cochrane Database Syst Rev* 2021;**2021**, DOI: 10.1002/14651858.CD015045.

Mohammed N, El-Hendawy S, Alsamin B *et al.* Integrating Application Methods and Concentrations of Salicylic Acid as an Avenue to Enhance Growth, Production, and Water

Use Efficiency of Wheat under Full and Deficit Irrigation in Arid Countries. *Plants* 2023;**12**, DOI: 10.3390/plants12051019.

Monteiro E, Gonçalves B, Cortez I *et al.* The Role of Biostimulants as Alleviators of Biotic and Abiotic Stresses in Grapevine: A Review. *Plants* 2022;**11**:1–18.

Nett RS, Lau W, Sattely ES. *Discovery and Engineering of Colchicine Alkaloid Biosynthesis*., 2020.

Nett RS, Sattely ES. Total Biosynthesis of the Tubulin-Binding Alkaloid Colchicine. *J Am Chem Soc* 2021;**143**:19454–65.

Nidorf SM, Fiolet ATL, Mosterd A *et al.* Colchicine in Patients with Chronic Coronary Disease. *N Engl J Med* 2020;**383**:1838–47.

Oraei M, Panahirad S, Zaare-Nahandi F *et al.* Pre-véraison treatment of salicylic acid to enhance anthocyanin content of grape (Vitis vinifera L.) berries. *J Sci Food Agric* 2019;**99**:5946–52.

Pertea M, Pertea GM, Antonescu CM *et al.* StringTie enables improved reconstruction of a transcriptome from RNA-seq reads. *Nat Biotechnol* 2015;**33**:290–5.

Qing Z, Liu J, Yi X *et al.* The chromosome-level Hemerocallis citrina Borani genome provides new insights into the rutin biosynthesis and the lack of colchicine. *Hortic Res* 2021;**8**:0–9.

Richette P, Bardin T. Colchicine for the treatment of gout. *Expert Opin Pharmacother* 2010;**11**:2933–8.

Robinson KP, Chan JJ. Colchicine in dermatology: A review. *Australas J Dermatol* 2018;**59**:278–85.

Schattner A. Colchicine – new horizons for an ancient drug. Review based on the highest hierarchy of evidence. *Eur J Intern Med* 2022;**96**:34–41.

Shoji T, Yuan L. ERF Gene Clusters: Working Together to Regulate Metabolism. *Trends Plant Sci* 2021;**26**:23–32.

Siak J, Flint N, Shmueli HG *et al.* The Use of Colchicine in Cardiovascular Diseases: A Systematic Review. *Am J Med* 2021;**134**:735-744.e1.

Ślusarkiewicz-Jarzina A, Pudelska H, Woźna J *et al.* Improved production of doubled haploids of winter and spring triticale hybrids via combination of colchicine treatments on anthers and regenerated plants. *J Appl Genet* 2017;**58**:287–95.

Solak Y, Siriopol D, Yildiz A *et al.* Colchicine in Renal Medicine: New Virtues of an Ancient Friend. *Blood Purif* 2017;**43**:125–35.

Stander EA, Papon N, Courdavault V. Puzzling Out the Colchicine Biosynthetic Pathway. *ChemMedChem* 2021;**16**:621–3.

Sun X, Wang Z, Li X *et al.* Effects of Yucca schidigera extract on serum biochemical parameters, humoral immune response, and intestinal health in young pigeons. *Front Vet Sci* 2023a;**9**, DOI: 10.3389/fvets.2022.1077555.

Sun Y, Zhou M, Luo L *et al.* Metabolic profiles, bioactive compounds and antioxidant activity of rosehips from Xinjiang, China. *Lwt* 2023b;**174**:114451.

Tammu RM, Nuringtyas TR, Daryono BS. Colchicine effects on the ploidy level and morphological characters of Katokkon pepper (Capsicum annuum L.) from North Toraja, Indonesia. *J Genet Eng Biotechnol* 2021;**19**, DOI: 10.1186/s43141-021-00131-4.

Tang D, Chen M, Huang X *et al.* SRplot: A free online platform for data visualization and graphing. *PLoS One* 2023;**18**:e0294236.

Tang MN, Liu X Bin, Huang JL *et al.* Questioning and arguable research on edible Hemerocallis citrina containing colchicine. *Chinese Tradit Herb Drugs* 2016;**47**:3293–300.

Tian H, Yang FF, Liu CY *et al.* Effects of phenolic constituents of daylily flowers on corticosterone- and glutamate-treated PC12 cells. *BMC Complement Altern Med* 2017;**17**:1–12.

Traub HP. Colchicine poisoning in relation to hemerocallis and some other plants. *Science (80-)* 1949;**110**:686–7.

Tucuch-Haas CJ, Pérez-Balam J V., Díaz-Magaña KB *et al.* Role of salicylic acid in the control of general plant growth, development, and productivity. *Salicylic Acid A Multifaceted Horm* 2017:1–15.

Wu J, Cheng X, Kong B *et al.* In vitro octaploid induction of Populus hopeiensis with colchicine. *BMC Plant Biol* 2022;**22**:1–11.

Yan M, Mao J, Wu T *et al.* Transcriptomic Analysis of Salicylic Acid Promoting Seed Germination of Melon under Salt Stress. *Horticulturae* 2023;**9**, DOI: 10.3390/horticulturae9030375.

Yi GE, Robin AHK, Yang K *et al.* Exogenous methyl jasmonate and salicylic acid induce subspecies-specific patterns of glucosinolate accumulation and gene expression in Brassica oleracea L. *Molecules* 2016;**21**, DOI: 10.3390/molecules21101417.

Zavala-gómez CE, Rodríguez-deleón E, Bah MM *et al.* Effect of salicylic acid in the yield of ricinine in Ricinus communis under greenhouse condition. *Plants* 2021;**10**:1–10.

Zhang S. Recent Advances of Polyphenol Oxidases in Plants. *Molecules* 2023;**28**, DOI: 10.3390/molecules28052158.

Zhong J, Liang Y, Chen Y *et al.* Study and Experimental Validation of the Functional Components and Mechanisms of Hemerocallis citrina Baroni in the Treatment of Lactation Deficiency. *Foods* 2021;**10**:1863.

Figure captions

Figure 1. (A)–(F) Morphology of control plant (A, CK) and SA-treated plants. B-F represent different SA treatments of T1 (0.5 mg/L), T2 (1 mg/L), T3 (2 mg/L), T4 (4 mg/L), and T5 (6 mg/L), respectively. (G)-(N) Variation in plant height, leaf length, leaf width, chlorophyll content, alabastrum length, alabastrum wide, alabastrum weight, and yield, respectively, of CK and different SA treatments. Different later above bars indicate statistical difference at $p < 0.05$.

Figure 2. Variation in biochemical traits between the control (CK) and the optimal SA treatment, T3 (2 mg/L). (A) Catalase activity; (B) Superoxide dismutase activity; (C) Total phenols content; (D) Total flavone content; (E) Soluble protein content; (F) Soluble sugar content; (G) reduced ascorbic acid content; and (H) Colchicine content. Significant difference was set at $p < 0.05$. The *P*-value of each comparison is shown at the top of the bar graphs.

Figure 3. (A) Principal component analysis (PCA) of samples based on TPM values of all genes. (B) Number differentially expressed genes (DEGs) between CK and T3.

Figure 4. (A) KEGG annotation and enrichment results of DEGs between CK and T3. (B) Expression patterns of DEGs related to antioxidant system. (C) Expression patterns of DEGs related to phenylpropanoid biosynthesis. The key is located on the right-hand side in each case.

Figure 5. Expression patterns of DEGs related to flavonoid biosynthesis (A), and transcription factors/phytohormones (B). The key is located on the right-hand side in each case.

Figure 6. qRT-PCR validation of the RNA-seq data and potential candidate genes. *, **, ***, and **** indicate significantly different at $P < 0.05$, 0.01, 0.001, and 0.0001, respectively

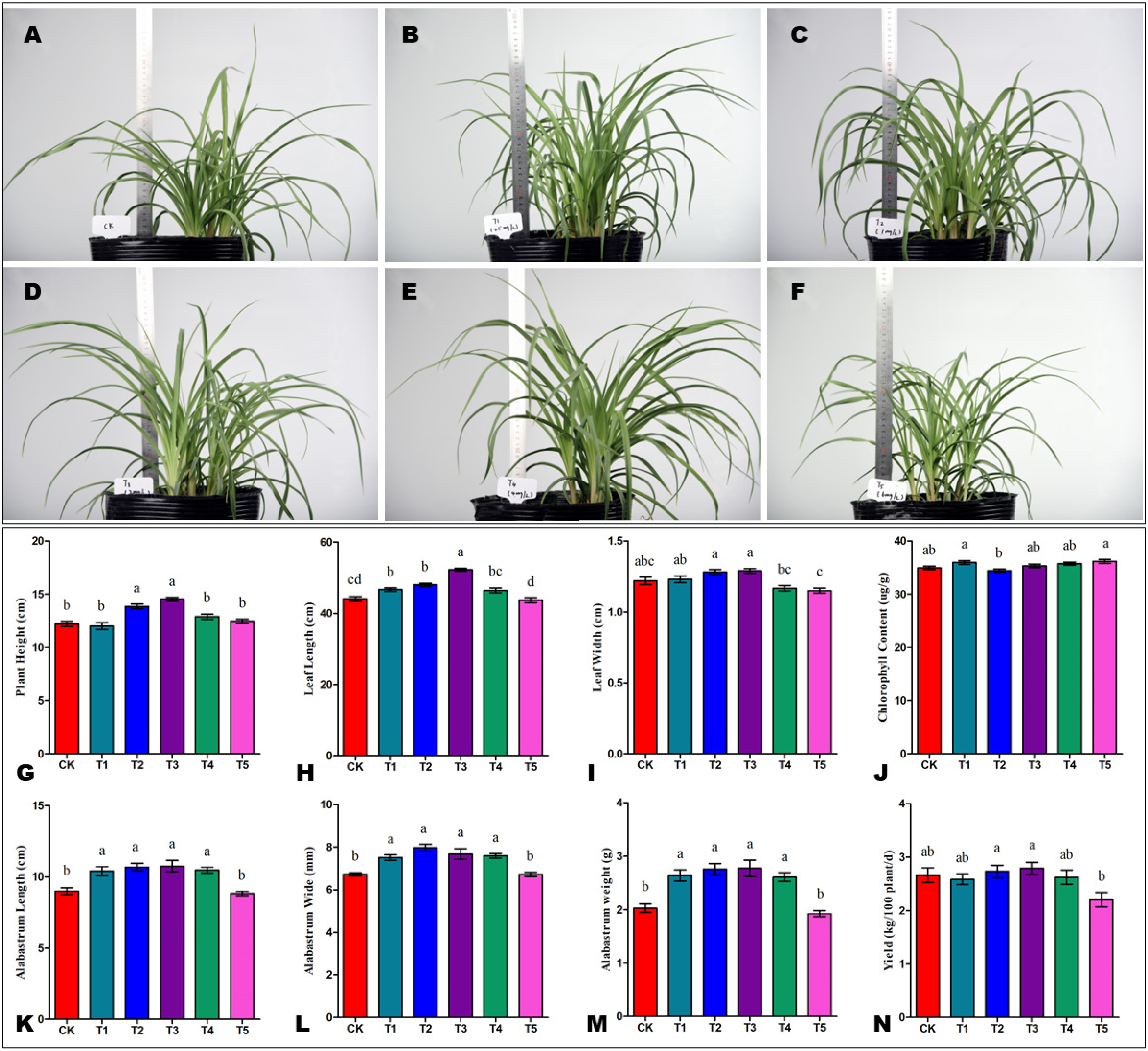
A
B
C
D
E
F
CK
G
H
I
J
K
L
M
N
Plant Height (cm)
Leaf Length (cm)
Leaf Width (cm)
Chlorophyll Content (ug/g)
Alabastrum Length (cm)
Alabastrum Wide (mm)
Alabastrum weight (g)
Yield (kg/100 plant/d)
CK T1 T2 T3 T4 T5

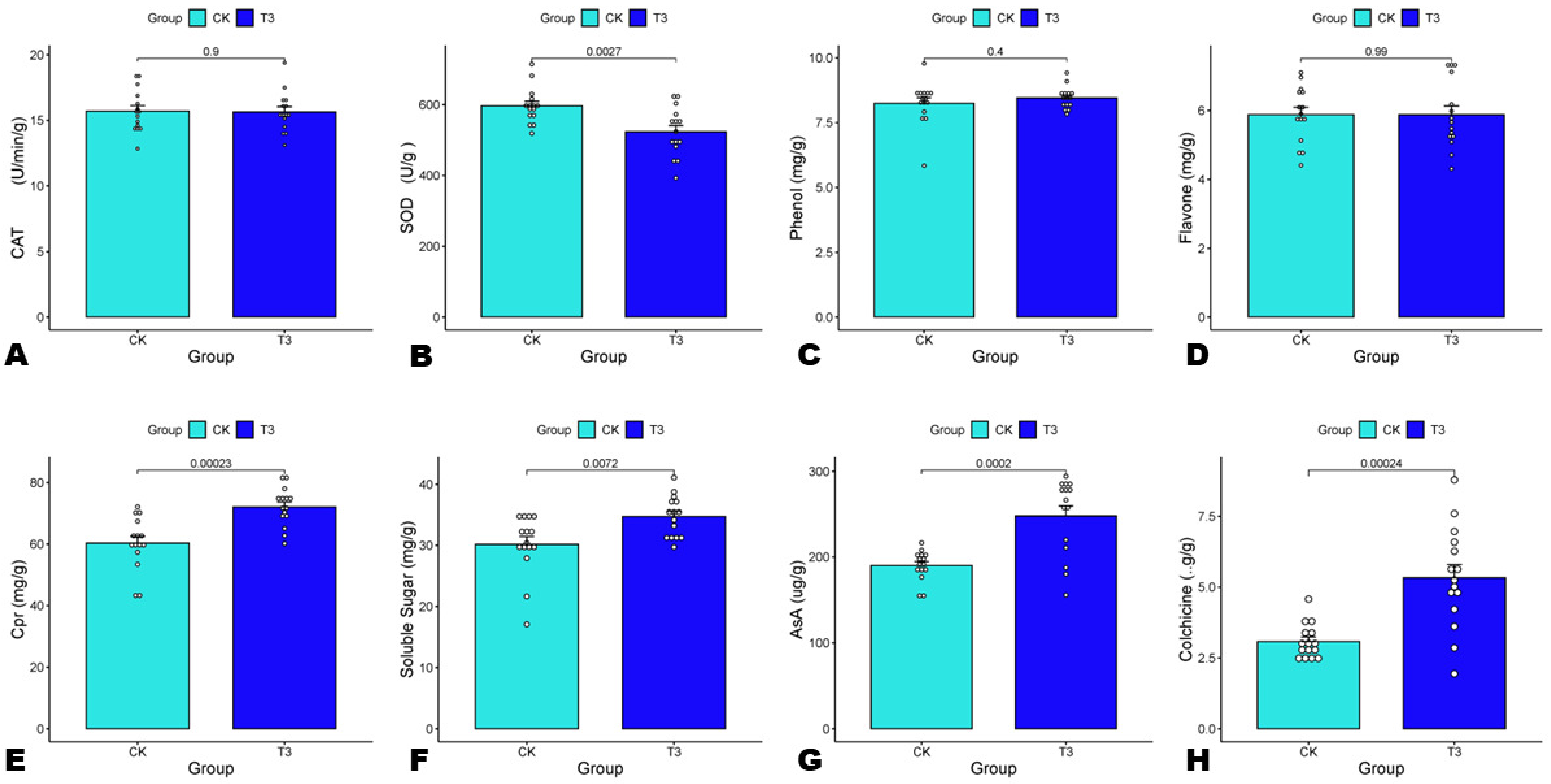
A
CAT (U/min/g)
0.9
B
SOD (U/g)
0.0027
C
Phenol (mg/g)
0.4
D
Flavone (mg/g)
0.99
E
Cpr (mg/g)
0.00023
F
Soluble Sugar (mg/g)
0.0072
G
AsA (ug/g)
0.0002
H
Colchicine (..g/g)
0.00024
Group
CK
T3

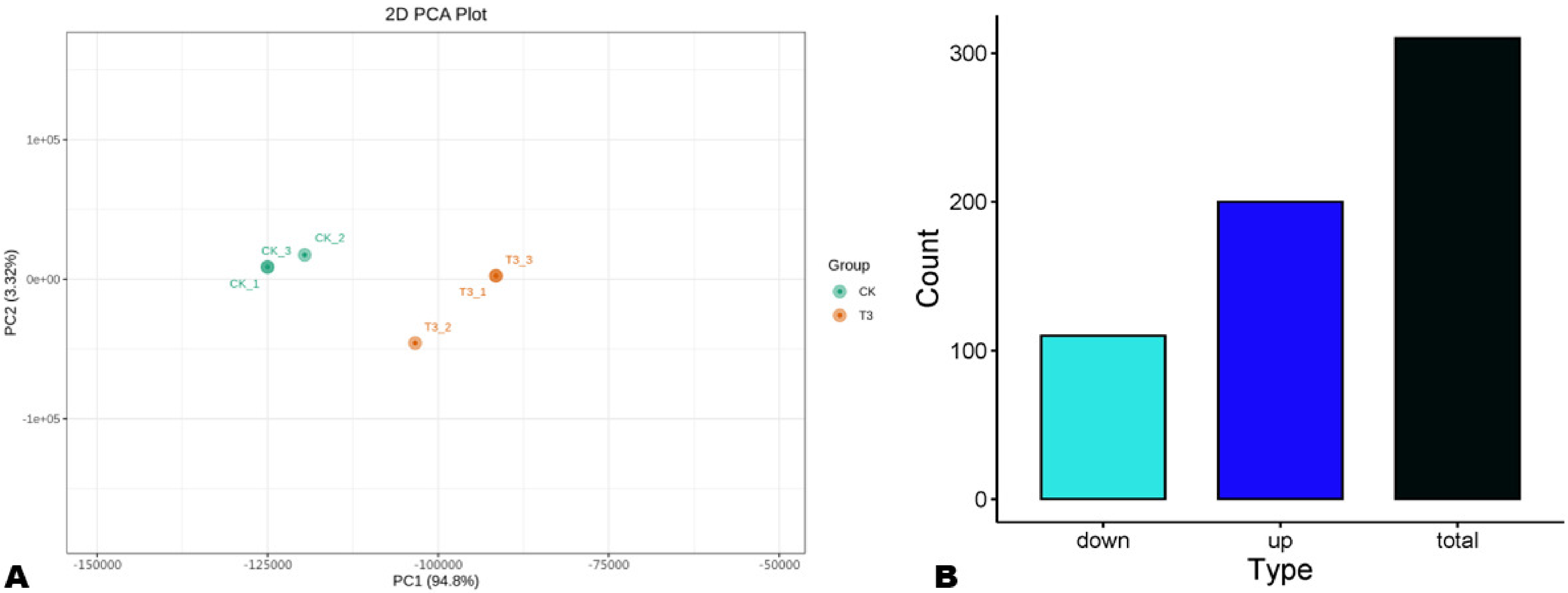
2D PCA Plot
PC2 (3.32%)
1e+05
0e+00
-1e+05
CK_1
CK_2
CK_3
T3_1
T3_2
T3_3
-150000
-125000
-100000
-75000
-50000
PC1 (94.8%)
Group
CK
T3
A
Count
300
200
100
0
down
up
total
Type
B

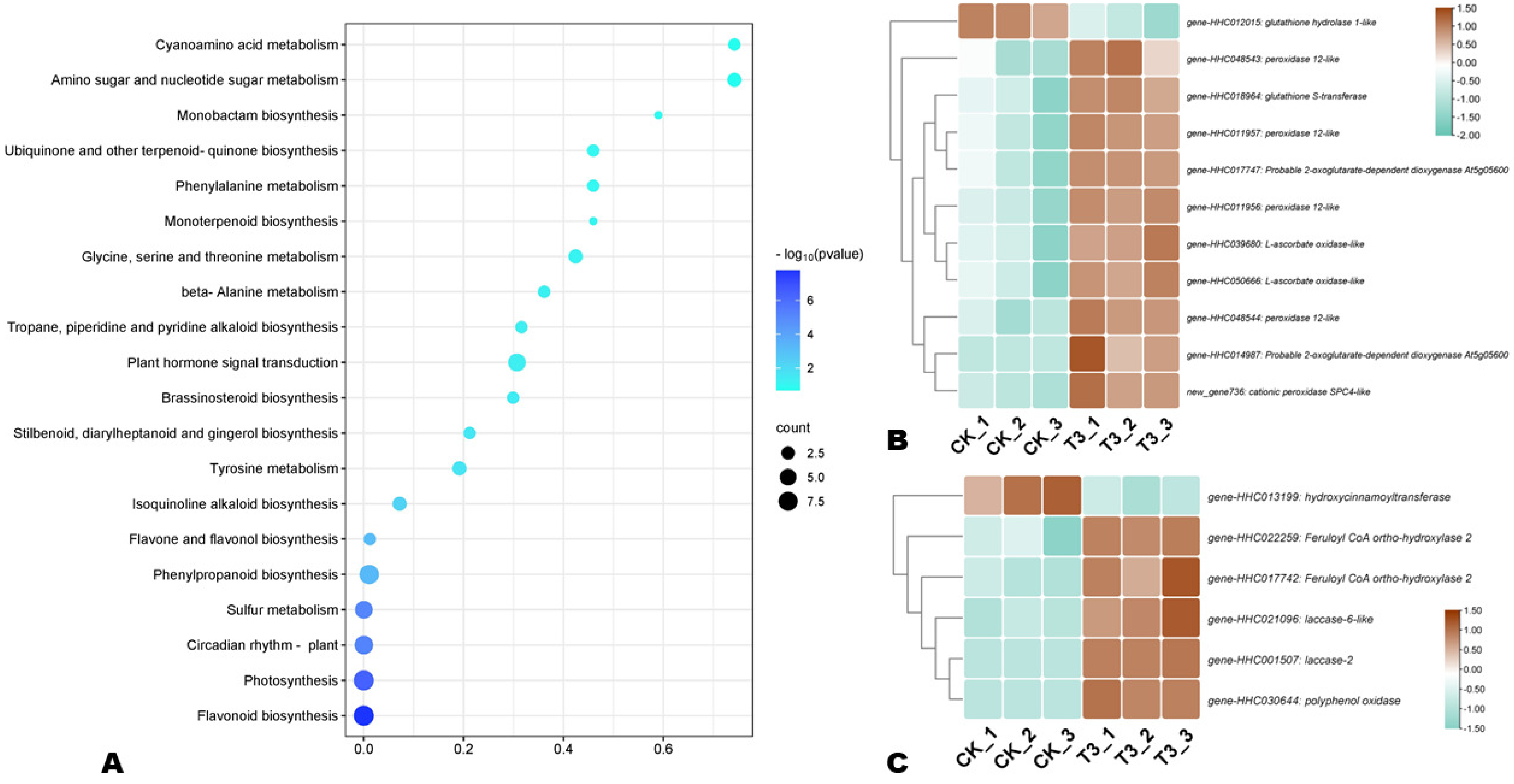
A
Cyanoamino acid metabolism
Amino sugar and nucleotide sugar metabolism
Monobactam biosynthesis
Ubiquinone and other terpenoid- quinone biosynthesis
Phenylalanine metabolism
Monoterpenoid biosynthesis
Glycine, serine and threonine metabolism
beta- Alanine metabolism
Tropane, piperidine and pyridine alkaloid biosynthesis
Plant hormone signal transduction
Brassinosteroid biosynthesis
Stilbenoid, diarylheptanoid and gingerol biosynthesis
Tyrosine metabolism
Isoquinoline alkaloid biosynthesis
Flavone and flavonol biosynthesis
Phenylpropanoid biosynthesis
Sulfur metabolism
Circadian rhythm - plant
Photosynthesis
Flavonoid biosynthesis
0.0
0.2
0.4
0.6
- log10(pvalue)
6
4
2
count
2.5
5.0
7.5
B
gene-HHC012015: glutathione hydrolase 1-like
gene-HHC048543: peroxidase 12-like
gene-HHC018964: glutathione S-transferase
gene-HHC011957: peroxidase 12-like
gene-HHC017747: Probable 2-oxoglutarate-dependent dioxygenase At5g05600
gene-HHC011956: peroxidase 12-like
gene-HHC039680: L-ascorbate oxidase-like
gene-HHC050666: L-ascorbate oxidase-like
gene-HHC048544: peroxidase 12-like
gene-HHC014987: Probable 2-oxoglutarate-dependent dioxygenase At5g05600
new_gene736: cationic peroxidase SPC4-like
CK_1
CK_2
CK_3
T3_1
T3_2
T3_3
1.50
1.00
0.50
0.00
-0.50
-1.00
-1.50
-2.00
C
gene-HHC013199: hydroxycinnamoyltransferase
gene-HHC022259: Feruloyl CoA ortho-hydroxylase 2
gene-HHC017742: Feruloyl CoA ortho-hydroxylase 2
gene-HHC021096: laccase-6-like
gene-HHC001507: laccase-2
gene-HHC030644: polyphenol oxidase
CK_1
CK_2
CK_3
T3_1
T3_2
T3_3
1.50
1.00
0.50
0.00
-0.50
-1.00
-1.50

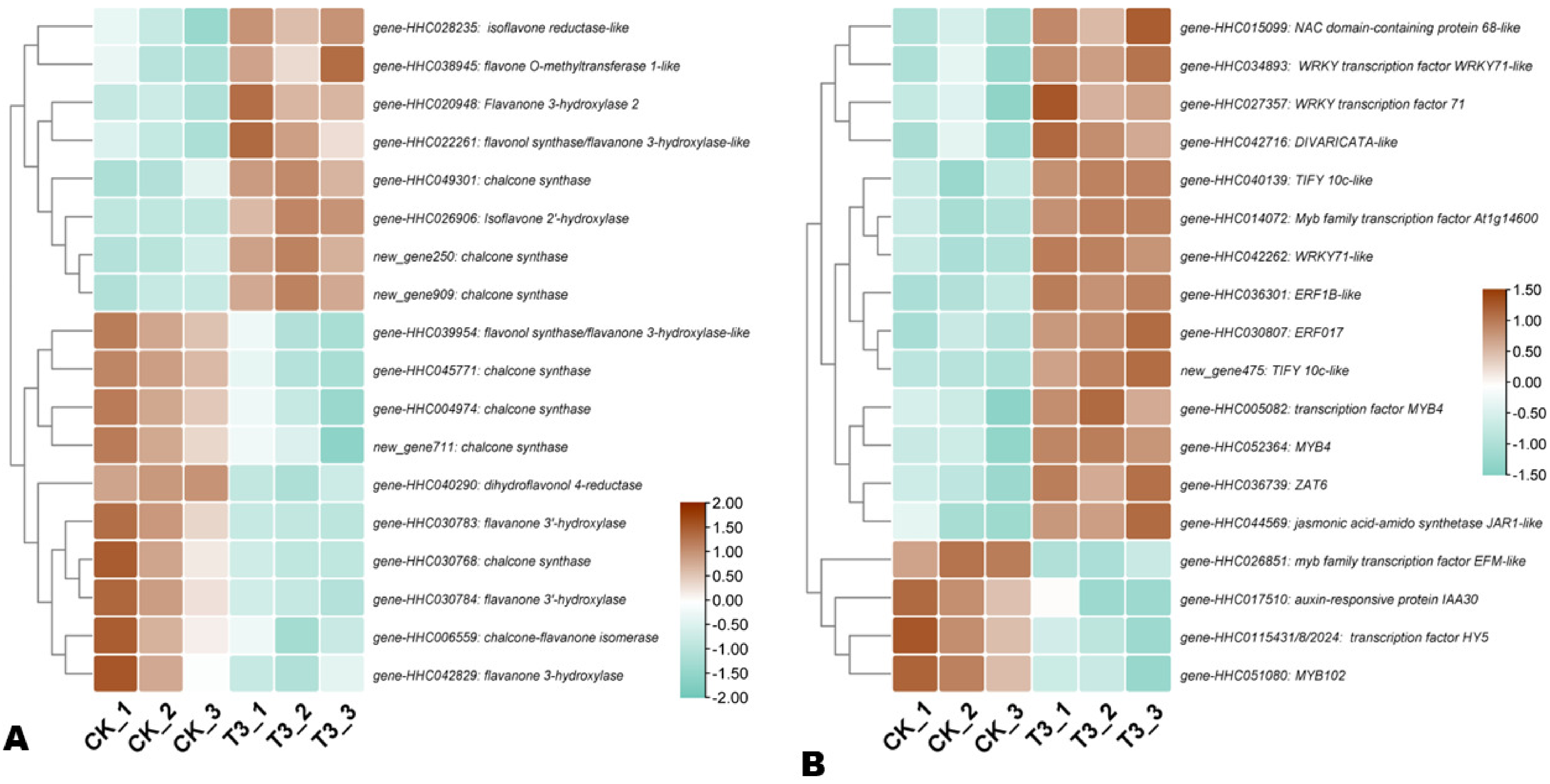
A
gene-HHC028235: isoflavone reductase-like
gene-HHC038945: flavone O-methyltransferase 1-like
gene-HHC020948: Flavanone 3-hydroxylase 2
gene-HHC022261: flavonol synthase/flavanone 3-hydroxylase-like
gene-HHC049301: chalcone synthase
gene-HHC026906: Isoflavone 2'-hydroxylase
new_gene250: chalcone synthase
new_gene909: chalcone synthase
gene-HHC039954: flavonol synthase/flavanone 3-hydroxylase-like
gene-HHC045771: chalcone synthase
gene-HHC004974: chalcone synthase
new_gene711: chalcone synthase
gene-HHC040290: dihydroflavonol 4-reductase
gene-HHC030783: flavanone 3'-hydroxylase
gene-HHC030768: chalcone synthase
gene-HHC030784: flavanone 3'-hydroxylase
gene-HHC006559: chalcone-flavanone isomerase
gene-HHC042829: flavanone 3-hydroxylase
2.00
1.50
1.00
0.50
0.00
-0.50
-1.00
-1.50
-2.00
CK_1
CK_2
CK_3
T3_1
T3_2
T3_3
B
gene-HHC015099: NAC domain-containing protein 68-like
gene-HHC034893: WRKY transcription factor WRKY71-like
gene-HHC027357: WRKY transcription factor 71
gene-HHC042716: DIVARICATA-like
gene-HHC040139: TIFY 10c-like
gene-HHC014072: Myb family transcription factor At1g14600
gene-HHC042262: WRKY71-like
gene-HHC036301: ERF1B-like
gene-HHC030807: ERF017
new_gene475: TIFY 10c-like
gene-HHC005082: transcription factor MYB4
gene-HHC052364: MYB4
gene-HHC036739: ZAT6
gene-HHC044569: jasmonic acid-amido synthetase JAR1-like
gene-HHC026851: myb family transcription factor EFM-like
gene-HHC017510: auxin-responsive protein IAA30
gene-HHC0115431/8/2024: transcription factor HY5
gene-HHC051080: MYB102
1.50
1.00
0.50
0.00
-0.50
-1.00
-1.50
CK_1
CK_2
CK_3
T3_1
T3_2
T3_3

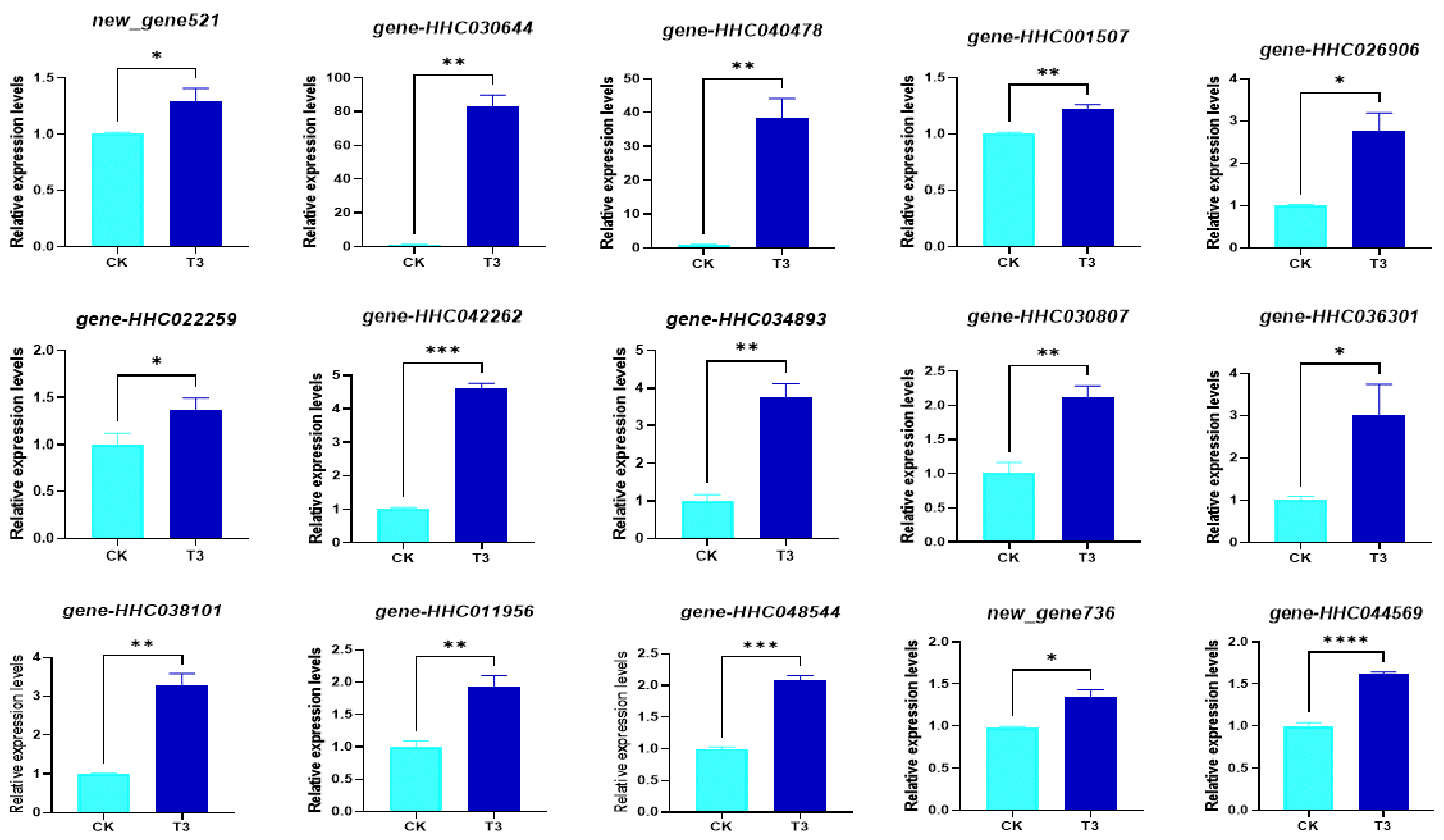
new_gene521
gene-HHC030644
gene-HHC040478
gene-HHC001507
gene-HHC026906
gene-HHC022259
gene-HHC042262
gene-HHC034893
gene-HHC030807
gene-HHC036301
gene-HHC038101
gene-HHC011956
gene-HHC048544
new_gene736
gene-HHC044569
Relative expression levels
CK
T3

Table 1. List of the potential candidate genes and their annotation

**Gene_ID**	**$\lvert$Log2FC$\rvert$**	**Nr Annotation**
new_gene521	13.263	NA
gene-HHC030644	8.367	XP_010910928.1^PREDICTED: polyphenol oxidase, chloroplastic^57.25%
gene-HHC040478	7.818	XP_021676207.1^germin-like protein subfamily 1 member 17^64.36%
gene-HHC001507	6.798	XP_020098212.1^laccase-2^79.38%
gene-HHC026906	6.268	AGI98133.1^cytochrome P450 CYP81^44.13%
gene-HHC022259	4.048	XP_020092748.1^protein DMR6-LIKE OXYGENASE 2^70.37%; Feruloyl CoA ortho-hydroxylase 2
gene-HHC042262	3.989	XP_020252240.1^WRKY transcription factor WRKY71-like^58.08%, Short=OsWRKY76^47.78%
gene-HHC034893	3.882	XP_020265728.1^WRKY transcription factor WRKY71-like^51.51%, Short=OsWRKY71^45.67%
gene-HHC030807	2.842	XP_004287662.1^ethylene-responsive transcription factor ERF017^53.15%
gene-HHC036301	2.422	XP_020248108.1^ethylene-responsive transcription factor 1B-like^64.25%, Short=AtERF1B
gene-HHC038101	1.883	XP_020257154.1^primary amine oxidase 1-like^75.23%
gene-HHC011956	1.753	XP_020107531.1^peroxidase 12-like^79.32%, Short=Atperox P12
gene-HHC048544	1.721	XP_020107531.1^peroxidase 12-like^81.99%, Short=Atperox P12
new_gene736	1.605	XP_020246461.1^cationic peroxidase SPC4-like^85.37%
gene-HHC044569	1.39	XP_020272199.1^jasmonic acid-amido synthetase JAR1-like^73.5%; Short=OsGH3-5
