# Supplementary material for: Salicylic acid modulates secondary metabolism and enhanced colchicine accumulation in long yellow daylily (Hemerocallis citrina)
Source: AoB Plants. 2024 May 21;16(4):plae029. doi: 10.1093/aobpla/plae029 (PMC11232463; doi:10.1093/aobpla/plae029)
Supplement: plae029_suppl_Supplementary_Materials [file plae029_suppl_supplementary_materials.zip › Supplementary Figures.pdf]

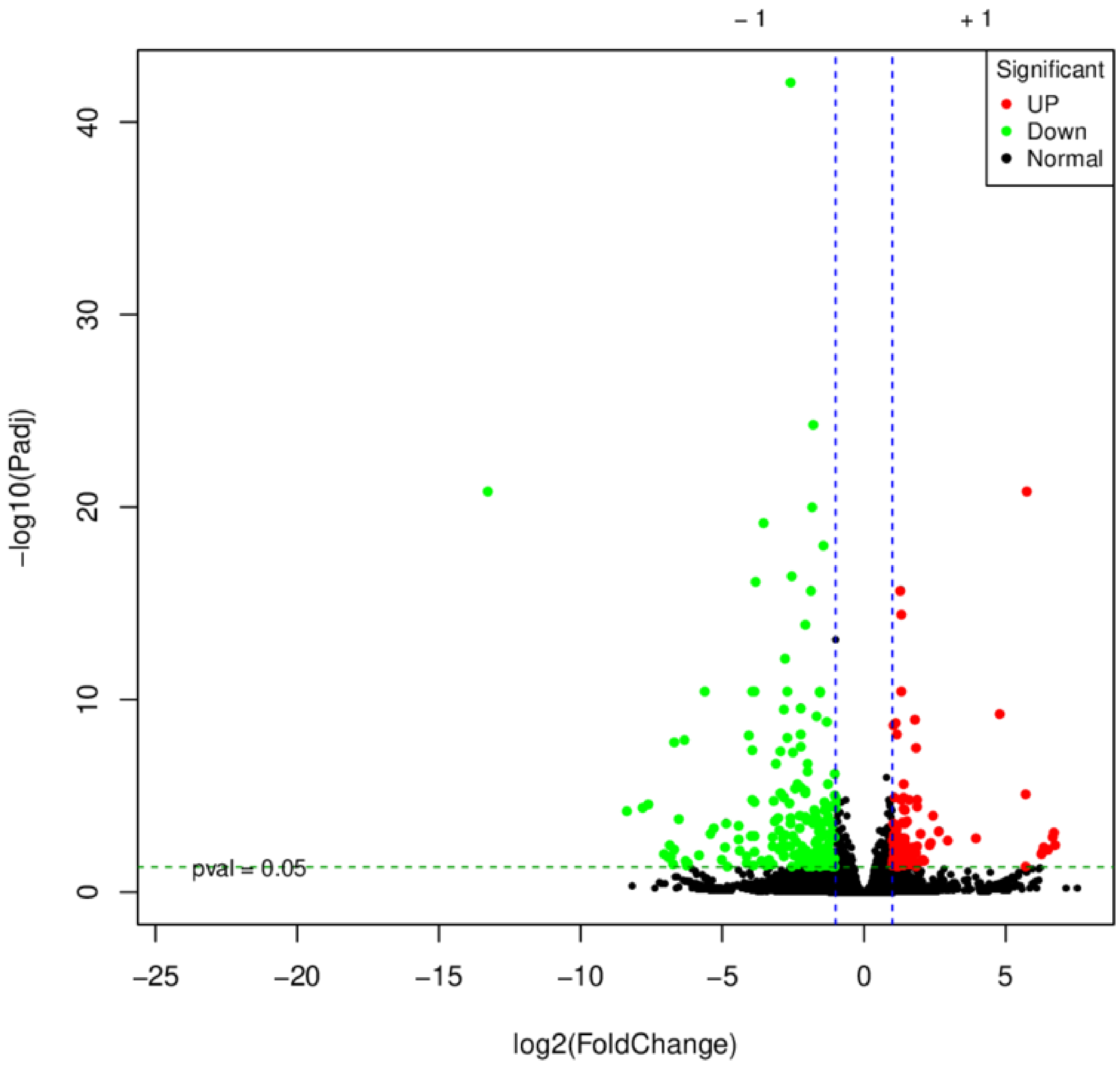

Figure S1. Volcano plot of differentially expressed genes (DEGs) between CK and T3. Green and red dots indicate up- and down-regulated genes in T3, respectively.

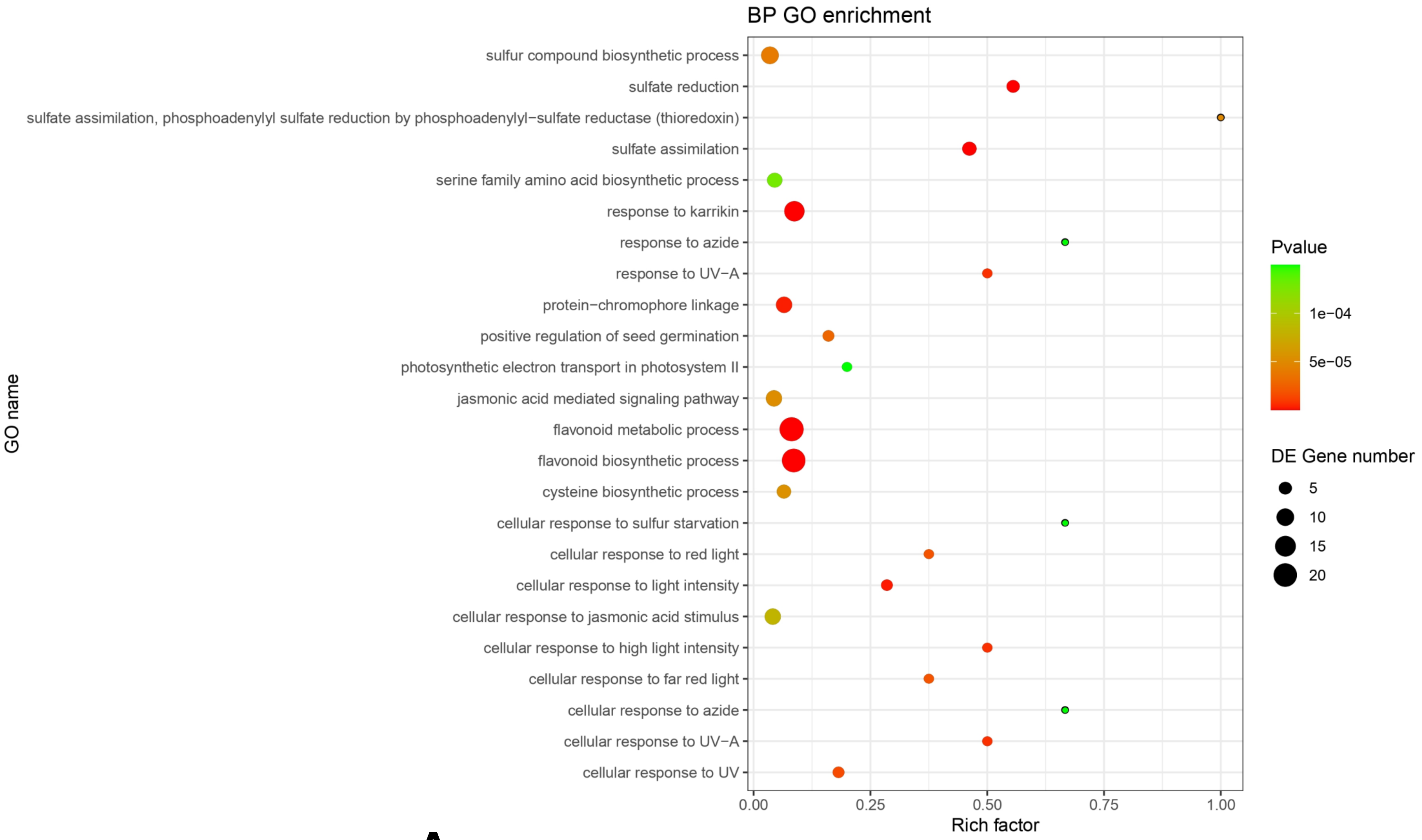

BP GO enrichment
sulfur compound biosynthetic process
sulfate reduction
sulfate assimilation, phosphoadenylyl sulfate reduction by phosphoadenylyl−sulfate reductase (thioredoxin)
sulfate assimilation
serine family amino acid biosynthetic process
response to karrikin
response to azide
response to UV−A
protein−chromophore linkage
positive regulation of seed germination
photosynthetic electron transport in photosystem II
jasmonic acid mediated signaling pathway
flavonoid metabolic process
flavonoid biosynthetic process
cysteine biosynthetic process
cellular response to sulfur starvation
cellular response to red light
cellular response to light intensity
cellular response to jasmonic acid stimulus
cellular response to high light intensity
cellular response to far red light
cellular response to azide
cellular response to UV−A
cellular response to UV
GO name
0.00
0.25
0.50
0.75
1.00
Rich factor
Pvalue
1e−04
5e−05
DE Gene number
5
10
15
20

A

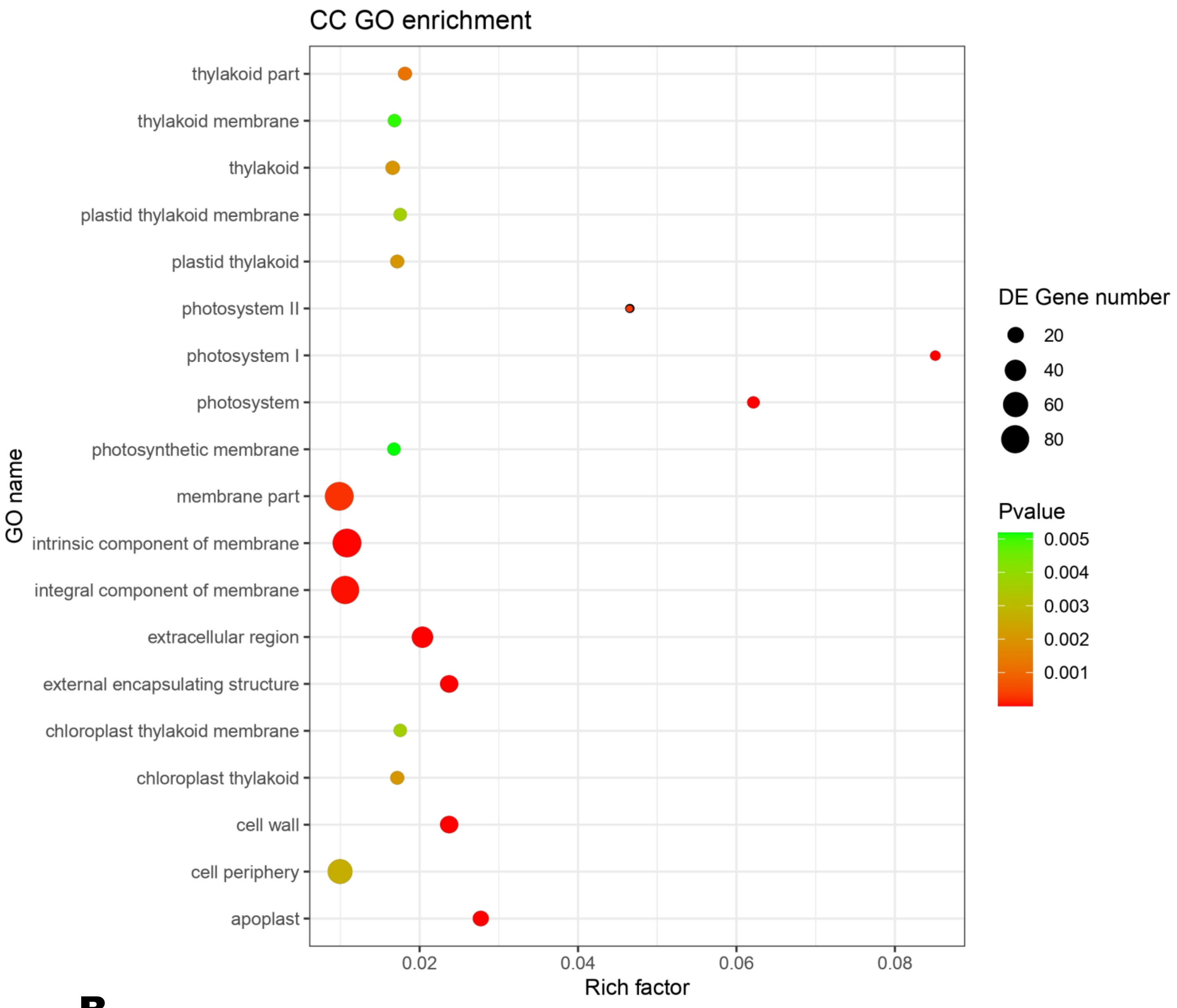
CC GO enrichment
GO name
thylakoid part
thylakoid membrane
thylakoid
plastid thylakoid membrane
plastid thylakoid
photosystem II
photosystem I
photosystem
photosynthetic membrane
membrane part
intrinsic component of membrane
integral component of membrane
extracellular region
external encapsulating structure
chloroplast thylakoid membrane
chloroplast thylakoid
cell wall
cell periphery
apoplast
0.02
0.04
0.06
0.08
Rich factor
DE Gene number
20
40
60
80
Pvalue
0.005
0.004
0.003
0.002
0.001

B

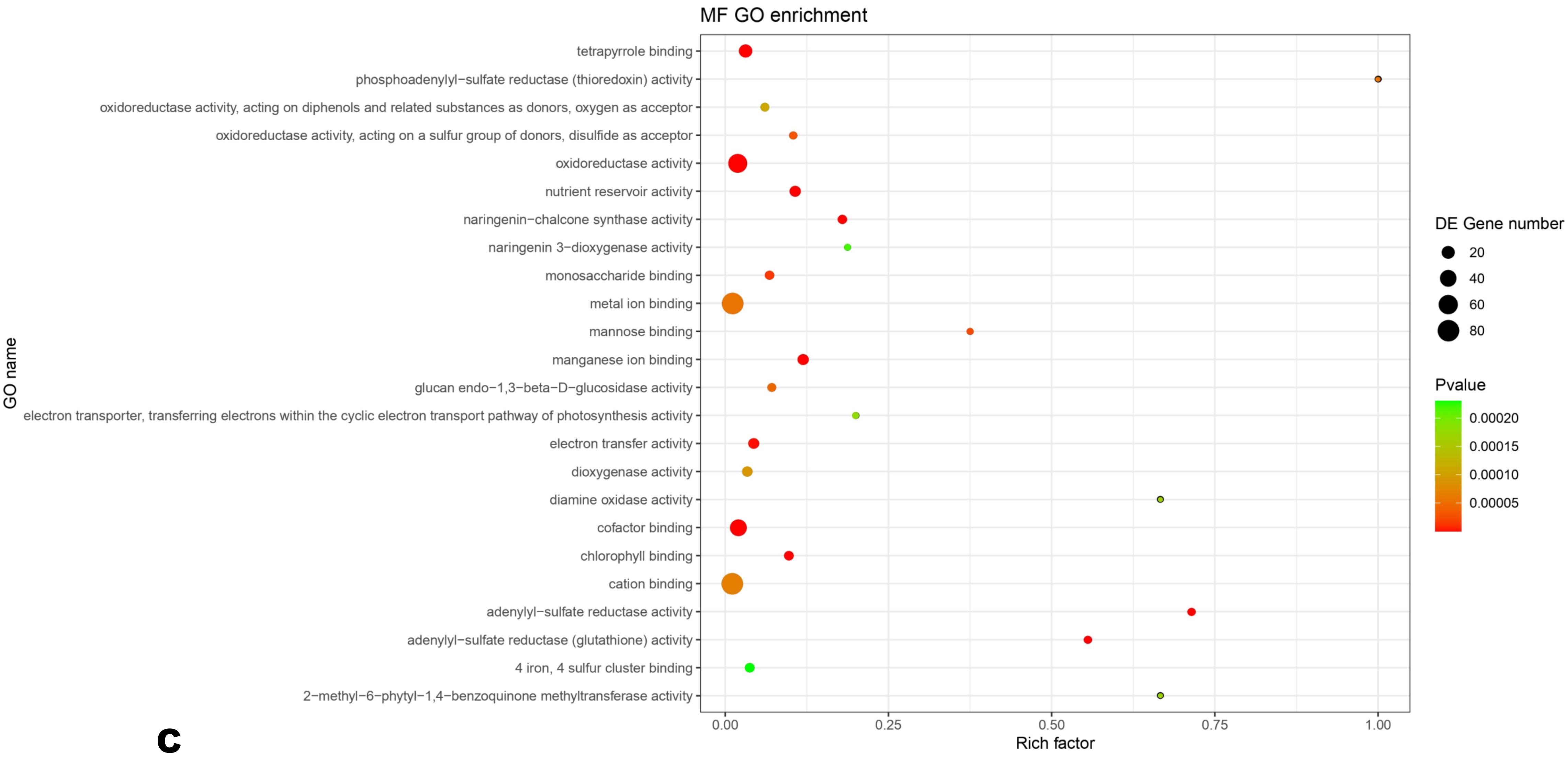

Figure S2. GO annotation and enrichment result of all DEGs. (A) Biological process. (B) Cellular component. (C) Molecular function.
