# Supplementary figures and images for: Salicylic acid modulates secondary metabolism and enhanced colchicine accumulation in long yellow daylily (Hemerocallis citrina)
Source: AoB Plants. 2024 May 21;16(4):plae029. doi: 10.1093/aobpla/plae029 (PMC11232463; doi:10.1093/aobpla/plae029)

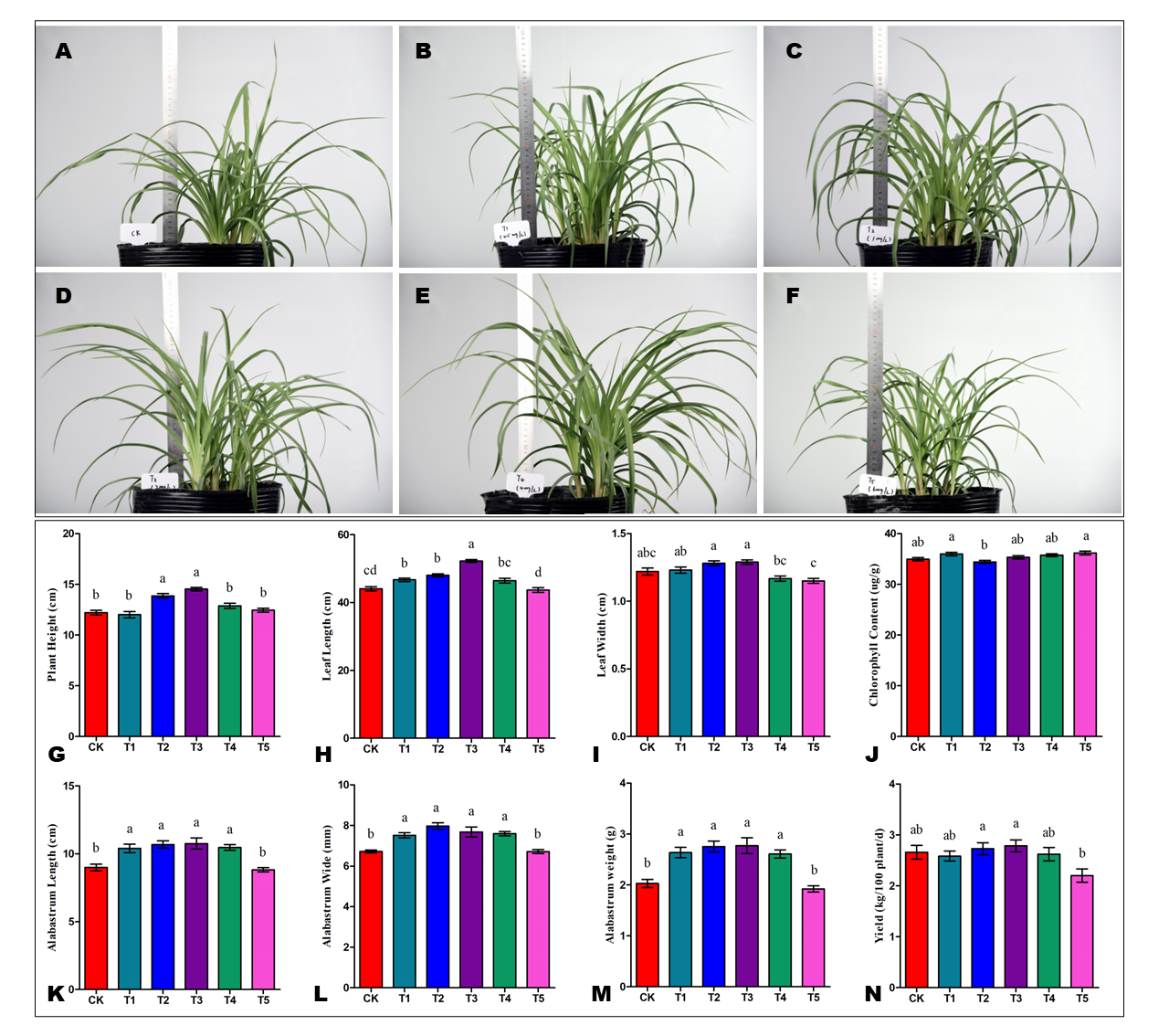

Supplement: plae029_suppl_Supplementary_Materials [file plae029_suppl_supplementary_materials.zip › aobplants-24039S1-f01-z-4c.png]

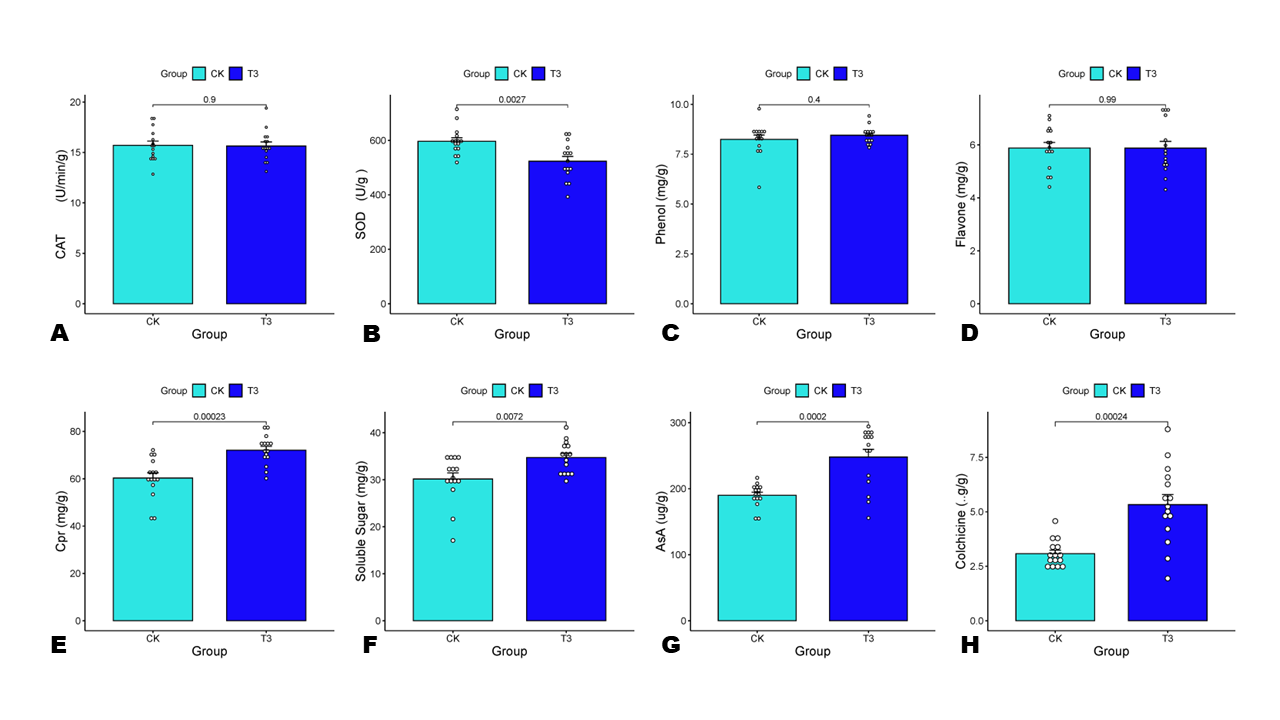

Supplement: plae029_suppl_Supplementary_Materials [file plae029_suppl_supplementary_materials.zip › aobplants-24039S1-f02-z-4c.png]

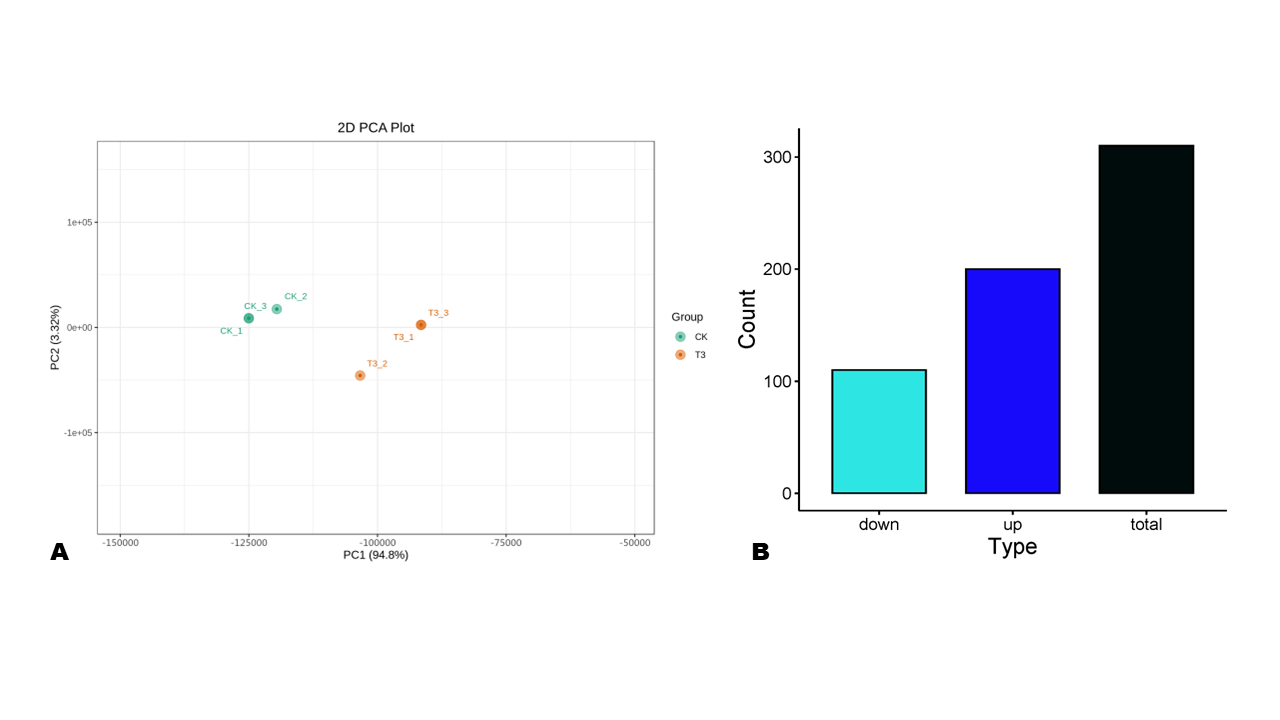

Supplement: plae029_suppl_Supplementary_Materials [file plae029_suppl_supplementary_materials.zip › aobplants-24039S1-f03-z-4c.png]

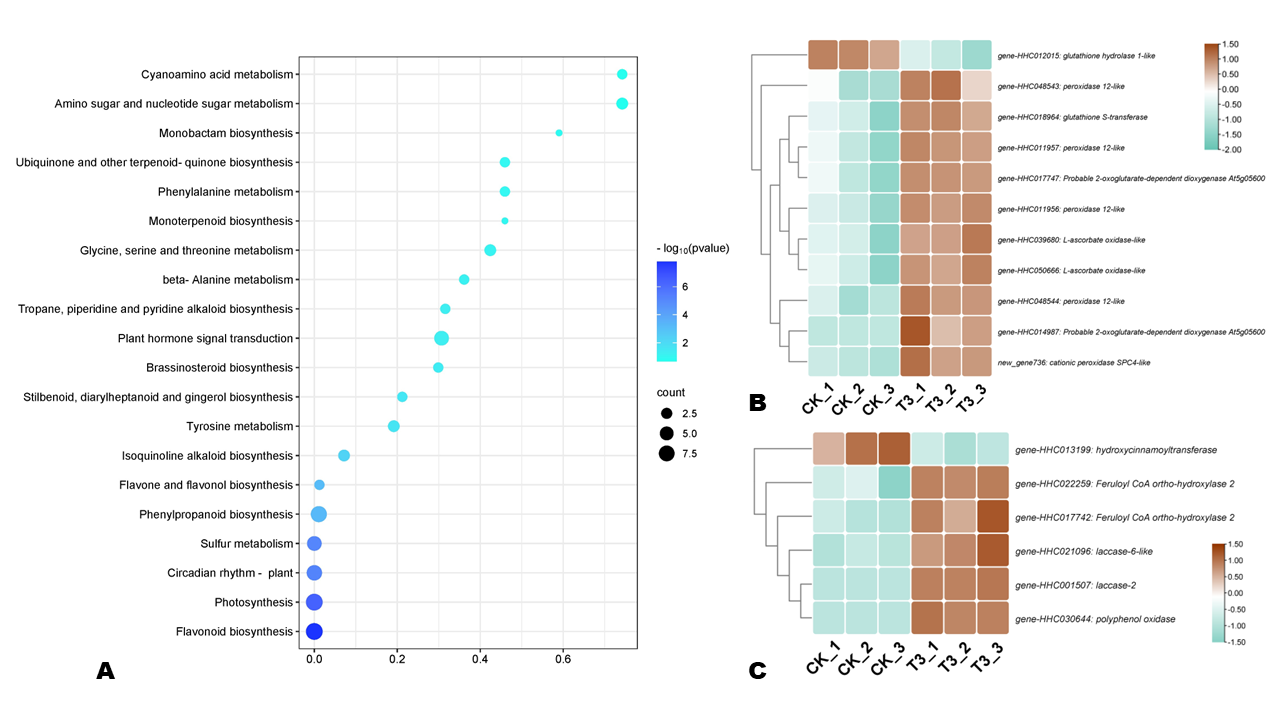

Supplement: plae029_suppl_Supplementary_Materials [file plae029_suppl_supplementary_materials.zip › aobplants-24039S1-f04-z-4c.png]

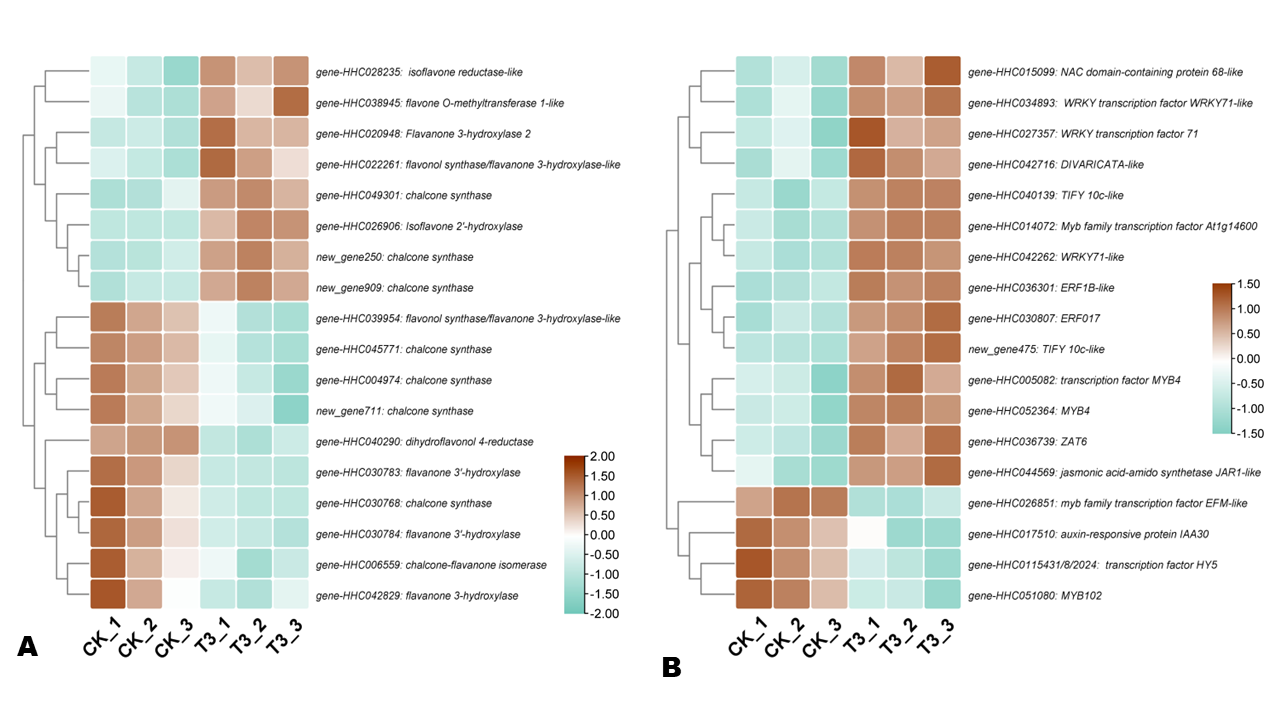

Supplement: plae029_suppl_Supplementary_Materials [file plae029_suppl_supplementary_materials.zip › aobplants-24039S1-f05-z-4c.png]

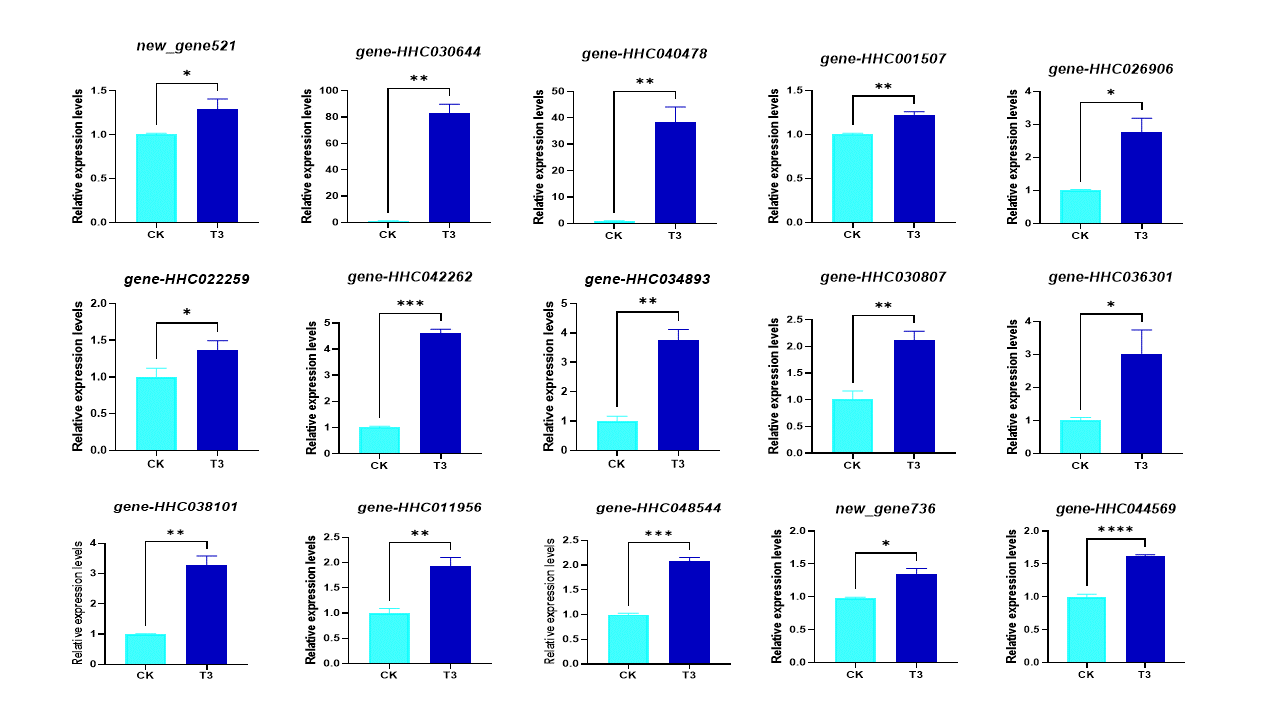

Supplement: plae029_suppl_Supplementary_Materials [file plae029_suppl_supplementary_materials.zip › aobplants-24039S1-f06-z-4c.png]
